# Explicitly Incorporating Prior Knowledge into Bayesian Optimization for Materials Design

**DOI:** 10.1021/acsomega.6c02171

**Published:** 2026-07-10

**Authors:** Hiroshi Aoki, Tomoyuki Miyao

**Affiliations:** 1 Graduate School of Science and Technology, 12708 Nara Institute of Science and Technology, 8916-5 Takayama-cho, Ikoma, Nara 630-0192, Japan; 2 Process Development Research Institute, Kao Corporation, 1334 minato, Wakayama-shi, Wakayama 640-8580, Japan; 3 Data Science Center, 12708 Nara Institute of Science and Technology, 8916-5 Takayama-cho, Ikoma, Nara 630-0192, Japan

## Abstract

We propose a novel Bayesian optimization (BO) method that leverages researchers’ knowledge of a linear relationship between experimental conditions **x** and a target property *y* to identify the optimal **x** with the fewest experiments. Knowledge is incorporated as the mean function μ­(**x**) of Gaussian process regression, and μ­(**x**) takes the form of a multivariate polynomial function of **x**, with appropriate prior distributions on the coefficients. These coefficients have prior distributions that reflect vague knowledge about the relationship between **x** and *y*, including the signs of the linear relationship. The posterior distribution of the coefficients is then obtained, and the acquisition functions in BO are derived using Markov chain Monte Carlo sampling to determine the next-to-be-tested **x** in the BO cycle. Through rigorous retrospective validations using mathematical simulation functions, a boiling-point data set, and a pigment data set, the method’s utility and limitations were revealed. Compared with previously proposed BO approaches, the proposed method identified better solutions with fewer experimental trials when prior knowledge aligned with the underlying relationship between **x** and *y*.

## Graphical abstract

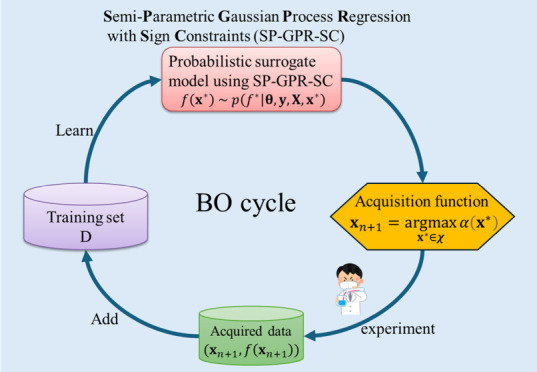

## 1 Introduction

Experiments are essential for designing novel functional materials. Cost and time constraints typically limit the number of experimental trials, necessitating methods to propose effective experimental conditions. To formalize this problem, one typically seeks to identify an experimental condition **x** that yields materials with the desired property *y* using the fewest experiments within a given experimental framework (e.g., equipment). For example, in the development of inorganic catalysis, the calcination temperature and duration are optimized to enhance catalytic activity. Likewise, in molecular discovery, compounds **x**s with desirable property *y* are investigated,^1^ where each compound is represented as a numerical vector called molecular descriptors. The problem of identifying the optimal experimental condition differs from standard functional optimization problems in that the underlying relationship between **x** and *y* is unclear and most likely unreachable; only a model-based relationship between **x** and *y* is available.

Bayesian optimization (BO) optimizes **x** by iteratively proposing **x**, measuring *y*, and updating the data set used to construct the model, even when the governing equation between **x** and *y* is nontrivial.^2^ BO methods fall under black-box optimization.^3^ BO techniques have been reported to identify better solutions with fewer experimental trials than other global optimization methods for several benchmark functions.^4^ In materials design, BO techniques have been extensively utilized due to the nature of the problems. Applications include phase-change memory material design,^5^ thin-film materials optimization,^6^ heterogeneous electrocatalyst identification,^7^ and nanoporous material design.^8^

In a standard BO procedure, when data pairs (**x**, *y*) are provided, a surrogate function mapping **x** to *y* is constructed using Gaussian process regression (GPR) to estimate the conditional probability distribution *y* given **x**: *p*(*y*|**x**). This conditional distribution is used to evaluate an acquisition function, which is optimized over **x** and typically yields a higher value for a better **x** than the current best **x**. Commonly used acquisition functions include the probability of improvement (PI),^9^ the expected improvement (EI),^2^ the upper confidence bound (UCB),^10^ and the predictive entropy search (PES).^11^ Maximizing an acquisition function yields a candidate **x**; **x** is tested in the next experiment, and the corresponding *y* is obtained. Repeating this process is expected to find the optimal **x** with fewer experimental trials than not using the BO methods.

One widely known issue in BO is the “cold start” problem.^12,13^ This problem states that naively applying BO methods without prior knowledge requires costly *y* values for a diverse set of **x** values, thereby diminishing the BO method’s advantage of reducing the number of experiments. To alleviate this problem, transfer learning methods have been proposed that leverage knowledge from similar experimental systems (source domain) to optimize the current experimental system (target domain). These transfer learning methods can be divided into two categories: data-point- and knowledge-based transfer.

In the first category, data points from the source domain are integrated into the target domain when building a GPR model. For example, a method was proposed to standardize the objective variables across both domains,^14^ adjust noise in both domains,^12,15^ emphasize differences in objective variables between the two domains, and correct the source-domain data based on these differences.^15^ Furthermore, using ranking instead of nominal values was proposed to ensure rigorous scaling of the objective variable,^16^ and a simple multitask GPR was employed to integrate the two domains.^13^ Information from the source domain about the global minima can also be incorporated into the probability density distribution to calculate an acquisition function in the target domain.^17^

In the second category, knowledge of a similar experimental system can be used as a constraint for the GPR surrogate function in BO. For example, the functional form of GPR models can be restricted to additive (i.e., the sum of models for individual dimensions) by using additive kernels.^18,19^ . This method exploits prior knowledge of the variables involved in the causal relationships, but not of the function itself. Furthermore, functional constraints have been incorporated into the GPR structure as long as the constraints can be represented by (partial) derivatives of the function,^20,21^ including monotonic increasing,^22^ unimodality,^23^ and U- and S-shapes.^24^

In materials and molecular design, experimental researchers often have prior knowledge of the relationship between **x** and *y*, which is often linear: an increase in **x** is associated with an improvement (or deterioration) in *y*. This prior knowledge may be inaccurate and vague, as subsequent data points may update it. The functional form restriction described above may be too strict for incorporating prior knowledge.

One simple method to incorporate prior knowledge of a linear relationship between **x** and *y* is to use semiparametric GPR (SP-GPR).^25^ SP-GPR assumes a multivariate linear regression (MLR) form for the mean function of a GPR model, and the prior distribution of the regression coefficients is assumed to be Gaussian. Although this approach can analytically derive the posterior distribution of the coefficients, more flexible forms of the coefficient priors, such as a uniform distribution that takes only positive values, are required to represent researchers’ knowledge about the target material.

Here, we propose a novel BO method that leverages researchers’ knowledge of a linear relationship between **x** and *y* to identify the optimal experimental condition **x** with the fewest experiments. The proposed GPR is SP-GPR with sign constraints, termed SP-GPR-SC. Knowledge is incorporated into the mean function μ­(**x**) of the GPR: μ­(**x**) is a multivariate polynomial in **x,** with an appropriate prior distribution on its coefficients. These coefficients have prior distributions that reflect vague knowledge about the relationship: the signs of the linear relationship between **x** and *y*. The posterior distribution of the coefficients is then obtained, and the acquisition function in BO is derived using Markov Chain Monte Carlo (MCMC) sampling to determine the next-to-be-tested **x** in the BO cycle.

As a proof-of-concept study, we conducted BO trials for three types of optimization problems: maximizing benchmark mathematical functions, performing molecular searches for a predefined boiling point (BP), and identifying pigment mixing ratios for a specified color. In the molecular search for a predefined BP, positive coefficients for molecular weight and hydrogen-bonding density are provided as prior knowledge. In identifying the mixing ratio of pigments for a specified color, effects on the light property are determined based on the nature of the pigments. Through rigorous retrospective validation by comparing with BO methods without prior knowledge, multivariate linear regression (MLR)-based optimization and intentionally incorporating inaccurate knowledge, the usefulness and limitations of the proposed method are discussed.

## 2 Materials and Methods

### 2.1 BO with Transfer Learning Framework

#### 2.1.1 General BO Workflow

The problem of finding the optimal experimental condition **x** in the *d*-dimensional domain $\chi \subseteq R^{d}$ that produces the maximum response (desired result) y∈R under the relation *y* = *F*(**x**) can be represented as

$$\underset{x\in \chi }{\mathrm{argmax}}F\left(x\right)$$ 1

Since the governing function *F*(**x**) is unreachable, efficient methods are required to find the optimal **x** without accessing *F*(**x**). The BO workflow begins with a set of initial experimental results {(**x**_*i*_, *y*_*i*_)}_*i* = 1···*n*_, from which a GPR model is constructed. The next experimental condition (point) **x**_*n*+1_ is proposed based on the acquisition function utilizing the GPR model, and the response *y*_*n*+1_ is obtained from the experiment. The obtained pair (**x**_*n*+1_, *y*_*n*+1_) is added to the existing data set, and the GPR model is updated. This process is repeated until the termination condition is satisfied.

#### 2.1.2 BO Workflow with SP-GPR-SC

The proposed BO workflow using SP-GPR-SC (BO (SP-GPR-SC)) is summarized in Figure 1, which extends the general BO workflow. The proposed workflow uses SP-GPR as a surrogate model architecture, and the sign constraints are introduced during sampling coefficient values from the prior. After the initial experimental data are obtained, researchers determine the incorporated knowledge of the relationship between **x** and *y* as sign constraints on the coefficients of the polynomial mean function of a GPR model, such as increasing x contributes to increasing y. Furthermore, the posterior distributions of the parameters, including the coefficients, are obtained via MCMC sampling. Using the obtained posterior predictive distribution, the acquisition function is evaluated, and the next experimental condition is determined. The resulting experimental outcome is added to the data set. This procedure is repeated until the terminal condition is satisfied.

**Figure 1:**
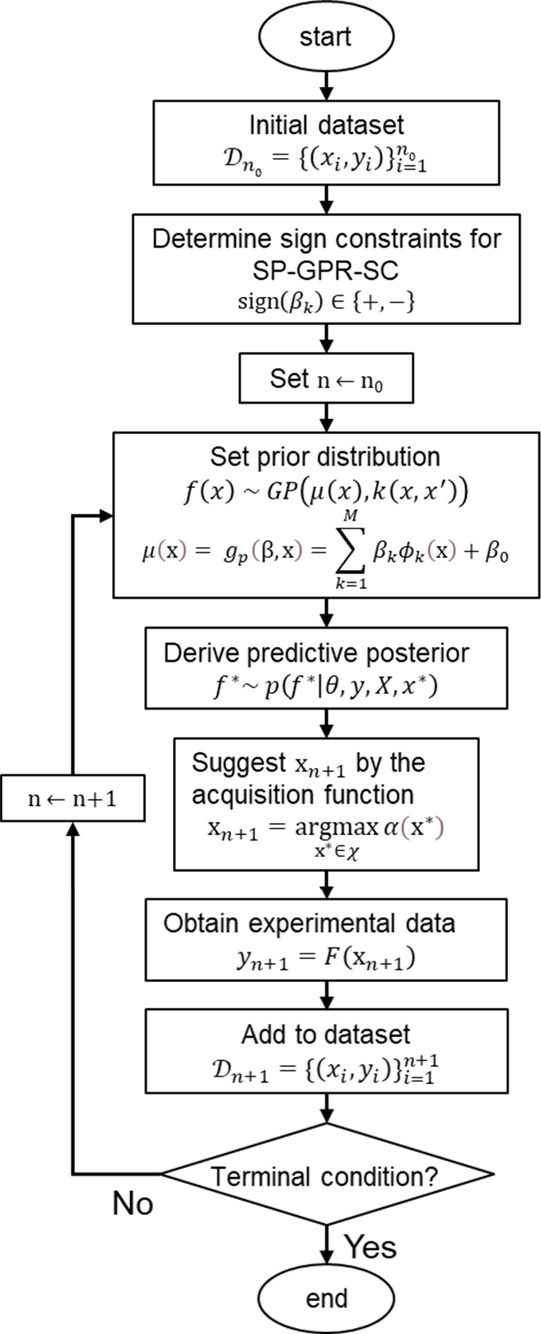
Workflow of Bayesian optimization (BO) using semiparametric GPR with sign constraints (BO (SP-GPR-SC)).

#### 2.1.3 Semiparametric GPR with Sign Constraints (SP-GPR-SC)

Our proposed SP-GPR-SC extends SP-GPR^25^ by incorporating prior knowledge via the coefficients of a linear structure, providing a prior distribution over the coefficients. The linear structure is used for the mean function of a GPR model. The observed variable *y* is regarded as being generated from the surrogate GPR model *f*(**x**) with a random variable noise ϵ.

y=f(x)+ϵ 2

ϵ follows a Gaussian distribution with mean 0 and variance σ_ϵ_^2^.

$$\epsilon ∼Ν(0,\sigma_{\epsilon }^{2})$$ 3

*f*(**x**) is modeled by GPR as follows:

$$f\left(x\right)∼\mathrm{GP}\left(\mu \left(x\right),k\left(x,x^{\prime }\right)\right)$$ 4

where, μ­(**x**) is the mean function and *k*(**x**, **x**’) is the kernel function. In SP-GPR, μ­(**x**) has a linear structure:

$$\mu \left(x\right)=g_{p}\left(\beta ,x\right)=\overset{M}{\underset{k=1}{\sum }}\beta_{k}\phi_{k}\left(x\right)+\beta_{0}$$ 5

Where ϕ_*k*_(**x**) is a basis function constructed from the original descriptors and may include feature transformations such as first- and second-order terms of *x*. $\beta =\left[\beta_{1},\beta_{2},\cdot \cdot \cdot ,\beta_{M},\beta_{0}\right]\in R^{M+1}$ is a set of coefficients, and a prior distribution is provided for **β**. The prior distribution can reflect knowledge about the relationship between ϕ_*k*_(**x**) and *y*, as explained in the next section.

The role of the sign constraints can be interpreted as introducing a directional bias into the response surface through the polynomial mean function. In a standard zero-mean GPR, the posterior mean tends to become zero in regions outside the training data domain. In contrast, SP-GPR-SC uses the polynomial mean function to encode the expected linear relationship between **x** and *y*. The sign constraints ensure that this directional trend is consistent with researchers’ knowledge.

#### 2.1.4 Knowledge Reflected on Prior Distribution of β

As a simple prior on β_*k*,_ which represents the relationship between ϕ_*k*_(**x**) and *y*, we focus on the positive or negative sign of it. Experimental researchers typically have a vague understanding of β_*k*_, such as that performance improves as the variable ϕ_*k*_(**x**) decreases. Knowledge of the sign of a coefficient can be expressed as a truncated normal distribution, where the negative or positive side of the normal distribution is truncated to force the variable to take the sign of the coefficient corresponding to the knowledge. In this study, we assumed that prior knowledge was extracted from a previously sampled data set, i.e., the initial data set for BO trials. Under this assumption, the mean of β_*k*_ was set to the *k*-th regression coefficient of the MLR model trained on the initial data set. In case the sign of the mean of β_k_ is inconsistent with the sign constraint (knowledge), β_k_ is still sampled from the truncated normal distribution normalized over the admissible region of the constraint because the untruncated normal distribution has nonzero density to that region. In this study, for all experiments, the standard deviation of β_*k*_ was set to 4, consistent with the appropriate range of *y* in the systems. The prior knowledge assumed for each case study will be explained in the Data set section.

#### 2.1.5 Kernel Functions

The kernel function in GPR (eq 4) determines the model’s smoothness. In this study, the automatic relevance determination (ARD)-based radial basis function (RBF) kernel function was used, which is defined as follows:

$$k\left(x_{i},x_{j}|c,\rho ,\sigma_{z}\right)=c^{2}\exp\left(-\frac{1}{2}\overset{D}{\underset{d=1}{\sum }}\frac{1}{\rho_{d}^{2}}{\left(x_{i,d}-x_{j,d}\right)}^{2}\right)+\delta_{i,j}\sigma_{z}^{2}$$ 6

The prior distributions for the ARD-based RBF kernel function are *c* ∼ Normal­(0, 1), ρ_*d*_ ∼ InvGamma­(5, 5), and σ_*z*_ ∼ Normal­(0, 1), where InvGamma represents the inverse gamma distribution. δ_*i*, *j*_ represents the Kronecker delta, which is defined as

$$\delta_{i,j}=\{\begin{array}{c} 1,i=j\\ 0,i\neq j \end{array}$$ 7

#### 2.1.6 Predictive Distribution of y

When the parameter set **θ** = [**β** ^*T*^, *c*, **ρ** ^*T*^, σ_*z*_^2^, σ_ϵ_^2^]^*T*^ is given, the prior distribution *p*(**y** | **θ,X**) of the sample data points **y** is a multivariate normal distribution, as follows:

$$p\left(y|\theta ,X\right)∼N\left(G\left(X\right),K+\sigma_{\epsilon }^{2}I\right)$$ 8

where *G*(**X**) ***=*** [*g*_*p*_(**β**, **x**_1_), *g*_*p*_(**β**, **x**_2_), ···, *g*_*p*_(**β**, **x**_*n*_)]^T^, **X** represents a collection of **x**s corresponding to **y**, and **I** being the *n* × *n* identity matrix. The kernel matrix **K** is defined as follows:

$$Κ=\left[\begin{array}{ccc} k\left(x_{1},x_{1}\right) & \cdots & k\left(x_{1},x_{n}\right)\\ \vdots & \ddots & \vdots \\ k\left(x_{n},x_{1}\right) & \cdots & k\left(x_{n},x_{n}\right) \end{array}\right]$$ 9

The predicted distribution *f* ^*^ at a new data point x* can be obtained by making a joint distribution of **y** and f* as

$$p\left(y,f^{*}|\theta ,X,x^{*}\right)∼N\left(\left[\begin{array}{c} G\left(X\right)\\ g_{p}\left(\beta ,x^{*}\right) \end{array}\right],\left[\begin{array}{cc} K+\sigma_{\epsilon }^{2}Ι & k^{*}\\ k^{*T} & k^{**} \end{array}\right]\right)$$ 10

where k* **=** [*k*(x*, **x**_**1**_) *k*(x*, **x**_**2**_)···*k*(x*, **x**_***n***_)]^**T**^ and k** **=** *k*(x*, x*). Using the formula for conditional distributions of the normal distribution, the predictive distribution of f* can be expressed as follows:

$$p\left(f^{*}|\theta ,y,X,x^{*}\right)∼N\left(\mu_{n}\left(x^{*}\right),\sigma_{n}^{2}\left(x^{*}\right)\right)$$ 11

The predicted mean μ_*n*_(**x**) and variance σ_*n*_^2^(x*) are provided as follows:

$$\mu_{n}\left(x^{*}\right)=g_{p}\left(\beta ,x^{*}\right)+k^{*T}{\left[Κ+\sigma_{\varepsilon }^{2}Ι\right]}^{-1}\left(y-G\left(X\right)\right)$$ 12

$$\sigma_{n}^{2}\left(x^{*}\right)=k^{**}-k^{*T}{\left[Κ+\sigma_{\varepsilon }^{2}Ι\right]}^{-1}k^{*}$$ 13

#### 2.1.7 Posterior Distribution of Regression Coefficients β

The posterior distribution of **β** and its point estimate are necessary for handling prior knowledge in a Bayesian manner. The posterior distribution of **β** after observing data set *D*_*n*_ = {**X**, **y**} is obtained following the Bayes’ theorem:

$$p\left(\beta |D_{n}\right)=\frac{p\left(D_{n}|\beta \right)p\left(\beta \right)}{\int p\left(D_{n}|\beta \right)p\left(\beta \right)d\beta }$$ 14

The posterior predictive distribution can be obtained by merging the posterior **θ,** which provides a fully Bayesian treatment of the model.

$$p\left(f^{*}|D_{n}\right)=\int p\left(f^{*}|\theta ,D_{n},x^{*}\right)p\left(\theta |D_{n}\right)\mathrm{d\theta}$$ 15

Since the posterior and the posterior predictive distributions are analytically intractable, a sampling method is required. We use the Markov Chain Monte Carlo sampling (MCMC) method implemented in stan version 2.19.^26^ The MCMC sampling parameters were algorithm: No-U-Turn sampler (NUTS), chains: 1, iter: 1000, warmup: 200, seed: 123. The remaining parameters were set to their default values.

In this study, to evaluate and interpret the updated distribution of **β,** the posterior mean was derived and used as a point estimate. The validity of the posterior distribution of **β** was assessed by comparing it with the true **β** in a case study using simulation data sets.

#### 2.1.8 Acquisition Functions

In a BO workflow, the next test point **x**_n+1_ can be obtained so that it maximizes the acquisition function α using the posterior predictive distribution of *f* ^*^ (eq. 15) as

$$x_{n+1}=\underset{x^{*}\in \chi }{\mathrm{argmax}}\alpha \left(x^{*}\right)$$ 16

As a proof-of-concept, we tested two types of scenarios: maximizing *y* and achieving a specific value *y*_target._ In the case study in which the maximization of *y* is required, PI and EI are employed, defined as follows.

$$\alpha_{\mathrm{PI}}(x^{*})≔p(f^{*}\geq f^{\prime })$$ 17

$$\alpha_{\mathrm{EI}}(x^{*})≔E[\max\{0,f^{*}-f^{\prime }\}]$$ 18

where *f*’ is the current maximum value in the data set, and E represents the expectation operation. Since we used MCMC samples for posterior parameters, eqs 17 and 18 can be estimated by the following equations:

$$\alpha_{\mathrm{PI}}(x^{*})\approx \frac{1}{N}\sum_{i=1}^{N}1(f^{*}(\theta^{i},D_{n},x^{*})\geq f^{\prime })$$ 19

where **1** is the indicator variable, taking 1 when an input argument is true, otherwise 0,

$$\alpha_{\mathrm{EI}}(x^{*})\approx \frac{1}{N}\sum_{i=1}^{N}\max(0,f^{*}(\theta^{i},D_{n},x^{*})-f^{\prime })$$ 20

In the second case, to achieve the specific target value *f*_target_, the following EI is used.

$$\alpha_{\mathrm{EI}}(x^{*})≔E[\max\{0,∥f^{\prime }-f_{\mathrm{target}}∥-∥f^{*}-f_{\mathrm{target}}∥\}]$$ 21

where *f*’ is the current best (closest to *f*_target_)

### 2.2 Evaluation Metrics

To evaluate how good an identified solution is, the distance to the target value (DT) was used:

$$\mathrm{DT}=∥f^{\prime }-f_{\mathrm{target}}∥$$ 22

where *f*_target_ is the target value of a task (the maximum value in the range or a specific target value, depending on the task) and *f*’ is the current best. Smaller DT means higher performance in BO. For each scenario, optimization trials are repeated multiple times, and the average DT value (DT_average_) is reported instead. To fairly evaluate DT_average_ values among different methods, the Wilcoxon rank sum test was conducted.

### 2.3 Methods for Comparison

The proposed SP-GPR-SC is compared with a standard BO method using a GPR model with a mean function of 0 and SP-GPR (multivariate regression form as μ­(**x**) without introducing sign constraints). Furthermore, an MLR-based optimization method is employed as a control, which simply samples **x**_*n*+1_ exhibiting the best predicted *y* value.

### 2.4 Case Studies and Knowledge in BO Trials

As a proof-of-concept study, we prepared and used three types of data sets: (1) benchmark mathematical functions, (2) a BP data set, and (3) a pigment data set. For each data set and scenario, multiple BO trials were conducted, and the transition of DT_average_ was monitored.

#### 2.4.1 Benchmark Mathematical Functions

Table 1 shows the six benchmark functions *F*(**x**) with two-dimensional **x** as input. For Functions No.1–3, the dominant terms of *F*(**x**) are polynomial, and their coefficients are targets for knowledge incorporation, while Functions No.4 to 6 exhibit strong nonlinear relationships between **x** and y, difficult to model with a polynomial. The latter three functions serve as controls to measure the effect of incorrectly incorporating linear knowledge into nonlinear *F*(**x**). No. 1 is prepared by the authors, No. 2 is the Branin-Hoo function,^27^ and No. 3 is the Cosine function.^28^ Functions from No. 4–6 were taken from Ackley,^29^ Rastrigin,^30^ and Schwefel.^31^ Figure 2 shows the contours of *F*(**x**), with the target coordinates highlighted by red triangles. Function No. 4–6 were originally defined as minimization problems, and their signs were reversed to align the optimization direction with the other benchmark functions.

**Table 1:** Benchmark Functions and Domains

no.	*F*(*x*)	domain
1	2*x*_1_ + 2*x*_2_ – |(*x*_1_ – 2)(*x*_1_ – 8)| – |(*x*_2_ – 2)(*x*_2_ – 8)|	*x*_1_ ∈ [0,10]*x*_2_ ∈ [0,10]
2	$\left(x_{2}-5.1{x_{1}}^{2}/(4\pi^{2})+(5/\pi )x_{1}-6{)}^{2}+10+10(1-18\pi )\cos(x_{1}\right)$	*x*_1_ ∈ [−5,10]*x*_2_ ∈ [0,15]
3	$1.6x_{1}+1.6x_{2}-2.56{x_{1}}^{2}-2.56{x_{2}}^{2}+1.2+0.3\cos\left(3\pi \left(1.6x_{1}-0.5\right)\right)+0.3\cos\left(3\pi \left(1.6x_{2}-0.5\right)\right)$	*x*_1_ ∈ [−0.2,0.8]*x*_2_ ∈ [−0.2,0.8]
4	$20\exp\left[-0.2{\left({x_{1}}^{2}/2+{x_{2}}^{2}/2\right)}^{0.5}\right]+\exp\left\{\left(\cos\left(2\pi x_{1}\right)+\cos\left(2\pi x_{2}\right)\right)/2\right\}-20-e$	*x*_1_ ∈ [−32.7, 32.7] *x*_2_ ∈ [−32.7, 32.7]
5	$-20-\left({x_{1}}^{2}-10\cos\left(2\pi x_{1}\right)\right)-\left({x_{2}}^{2}-10\cos\left(2\pi x_{2}\right)\right)$	*x*_1_ ∈ [−5.1, 5.1] *x*_2_ ∈ [−5.1, 5.1]
6	–837.9658 + *x*_1_ sin (|*x*_1_|^0.5^) + *x*_2_ sin (|*x*_2_|^0.5^)	*x*_1_ ∈ [−500, 500] *x*_2_ ∈ [−500, 500]

**Figure 2:**
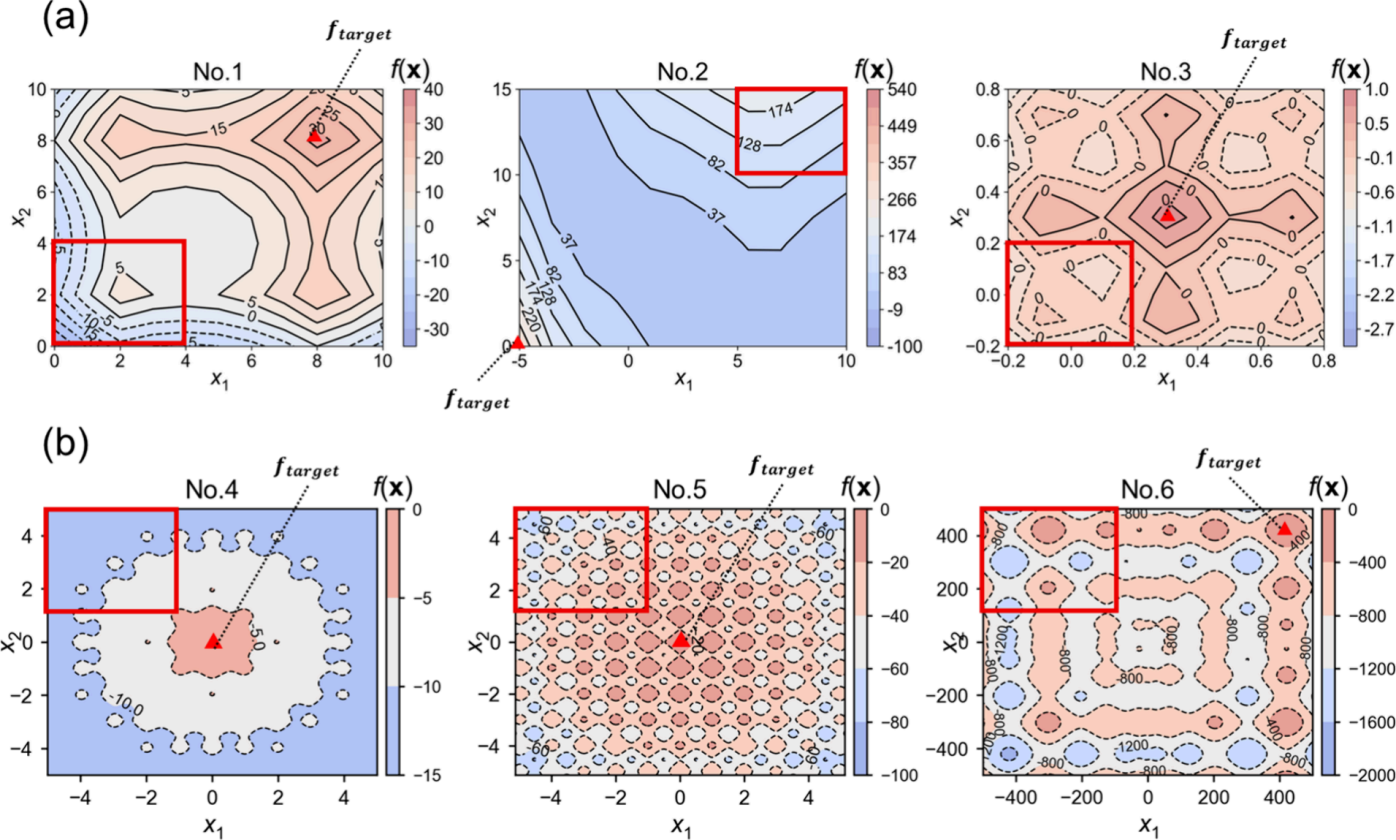
Contours of the six benchmark functions. *f*_target_ (red triangles), and initial data point regions (red rectangle) for the six benchmark functions are also shown. The three functions having dominant terms as polynomials are shown in (a), and the three functions exhibiting strong nonlinear relationships between **x** and y are shown in (b).

For each of the benchmark functions No.1, 2, and 3, the form of the mean function *g*_*p*_(**β**,**x**) and its prior *p*(**β**) as introduced knowledge is reported in Table 2. For function No.1, we assumed positive linear relationships between *x*_1_ and *y*, *x*_2_ and *y,* based on the ground-truth benchmark function terms. For functions No. 2 and No. 3, we assumed that linear relationships for *x*_1_ and *x*_2_, and their squared terms *x*_1^2^_ and *x*_2^2^_. The ground-truth values for **β** are estimated simply by focusing on the terms of the mean functions, as shown in Table 2. For functions No.4 to 6, a simple linear model *g*_p_(**β**,**x**) = β_1_ *x*_1_+β_2_ *x*_2_+β_0_ with the positive sign constraint for β_1_ and β_2_ was assumed as control.

**Table 2:** Assumed Knowledge of the Benchmark Functions^a^

no.	1			2							3				
*g*_*p*_(**β, x**)	β_1_ *x*_1_ + β_2_ *x*_2_ + β_0_			β_11_ *x*_1_ + β_21_ *x*_2_ + β_12_ *x*_1_^2^ + β_22_ *x*_2_^2^ + β_13_ *x*_1_^3^+ β_14_ *x*_1_^4^ + β_0_							β_11_ *x*_1_ + β_21_ *x*_2_ + β_12_ *x*_1_^2^ + β_22_ *x*_2_^2^ + β_0_				
parameter	β_1_	β_2_	β_0_	β_11_	β_21_	β_12_	β_22_	β_13_	β_14_	β_0_	β_11_	β_21_	β_12_	β_22_	β_0_
ground truth	2	2	0	–19	–12	4.08	1	–0.4	0.02	46	1.6	1.6	–2.6	–2.6	1.2
sign in prior	+	+		–	–	+	+	–	+		+	+	–	–	

a Black cells indicate no sign constraints.

For each benchmark function, an initial data set *D*_0_ = {**X**, **y**} is created by randomly sampling three data points from the red box in Figure 2. The BO iteration was repeated 20 times, and at each iteration, one **x** point is selected from a pool of unselected data points. This procedure was repeated 50 times, yielding 50 distinct BO trials, and the average DT value was calculated as the evaluation metric described above.

#### 2.4.2 BP Data Set

A data set of 5188 compounds with BPs measured under atmospheric pressure was used for BO^32^, trials. A BP histogram for the data set is provided in Figure S1 of the Supporting Information (SI). The BP ranges from 20 to 990K. The goal was to identify a compound with a specified BP, using the fewest experimental iterations. The independent variables **x** comprised 9 molecular descriptors derived from the compound SMILES strings. The descriptors listed in Table 3 were selected because they are associated with hydrogen bonding and molecular size, which affect the BPs of compounds. Because the relationship between BP and MW often appears exponential or power-law,^33^ MW was chosen as log­(MW), as in *x*_9_.

**Table 3:** Molecular Descriptors for the BP Dataset

variable	description
*x*_1_	number of hydrogen bond donors
*x*_2_	number of hydrogen bond acceptors
*x*_3_	number of heavy atoms (not hydrogen atoms)
*x*_4_	number of carbon atoms
*x*_5_	number of nitrogen atoms
*x*_6_	number of oxygen atoms
*x*_7_	log *P*: n-octanol–water partition coefficients
*x*_8_	topological polar surface area
*x*_9_	log(molecular weight)

For the BO experiment, we assumed two situations: using nonredundant and redundant descriptors. Unlike identifying the best experimental condition for a specific purpose, we can employ arbitrary descriptors (variables) for molecular discovery. We used only two variables to test the first situation: topological polar surface area (*x*_8_) and log­(Molecular weight) (*x*_9_). For the second situation, all 9 descriptors were used as **x**. In both situations, positive sign constraints were imposed on β_8_, corresponding to topological polar surface area, and β_9_, corresponding to log­(Molecular weight). It is well-known that BP is positively correlated with the strength of hydrogen bonding and molecular weight. Thus, these factors are incorporated into the prior distribution of the coefficients for the corresponding descriptors in *g*_*p*_(**β**,**x**) (β_8_ > 0 and β_9_ > 0).

In each scenario, *f*_target_ was set to a randomly selected BP value above 400 K, and 20 trials were conducted, each consisting of 20 cycles of BO. In each iteration, a single compound was selected using the acquisition function in Eq. 20. The initial data set consisted of three compounds with boiling points below 200 K.

#### 2.4.3 Pigment Data Set

A pigment combination can produce a color. Colors are represented in the CIELAB color space (L*, a*, b*), where L* represents lightness, a* the red-green component, and b* the yellow-blue component. The color of a mixture of pigments was determined by an in-house L*a*b* simulator (Section S1) based on the Kubelka–Munk theory.^34^ The L*, a*^,^ and b* distributions calculated by the simulator are shown in Figure S2. The simulator takes a pigment composition as input, and outputs the optical properties L*, a*, and b*. Parameters in the simulator were extracted from the report by Aoyama et al.,^35^ which is explained in detail in Section S1. The goal was to identify the pigment composition that yields a predefined color, and multiple objectives were incorporated into the acquisition function in eq 21. The extracted pigments and putative effects on the CIELAB parameters are shown in Table 4. The color categories in Table 4, such as red, were inferred by the authors from the raw material names, and coating images are also provided in the reference.^35^ Based on the categorized colors in Table 4 and their placement in the L*, a*, b* space (red takes a positive value for a*, green takes a negative value for a*, yellow takes a positive value for b*, and blue takes negative for b*^36^), the effect of each ingredient on color was summarized and is reported in Table 4. It should be noted that the reflected light appears white when no pigment is applied, and the whiteness decreases even when white pigment is added (ID3 in Table 4). Independent variables *x*_1_ to *x*_6_ are the concentrations [wt %] of the corresponding six pigments in Table 4. The SP-GPR-SC models were independently built for L*, a*, and b*, and the knowledge introduced in these models is summarized in Table 5. The intercept term shown in Table 5 is the measured reflectance of the base when no pigment is applied.

**Table 4:** Pigment Profile and the Pigment Effects on the CIELAB Parameters^a^

ID	pigment name	color category	direction effect		
			L*	a*	b*
1	Bengala	red	–	+	+
2	Inari yellow clay	yellow	–	+	+
3	Tahara white clay	white	–		
4	Lascaux black	black	–		
5	Italian green clay	green	–	–	
6	blue gray powder	blue	–	–	–

a Black cells indicate no sign constraints.

**Table 5:** Prior Knowledge of Color Pigments and L*a*b*^a^

target	L*							a*							b*						
*g*_*p*_(**β, x**)	$\overset{6}{\underset{k=1}{\sum }}\beta_{k}^{\left(L\right)}x_{k}^{\left(L\right)}+\beta_{0}^{\left(L\right)}$							$\overset{6}{\underset{k=1}{\sum }}\beta_{k}^{\left(a\right)}x_{k}^{\left(a\right)}+\beta_{0}^{\left(a\right)}$							$\overset{6}{\underset{k=1}{\sum }}\beta_{k}^{\left(b\right)}x_{k}^{\left(b\right)}+\beta_{0}^{\left(b\right)}$						
parameter	β_1_^(*L*)^	β_2_^(*L*)^	β_3_^(*L*)^	β_4_^(*L*)^	β_5_^(*L*)^	β_6_^(*L*)^	β_0_^(*L*)^	β_1_^(*a*)^	β_2_^(*a*)^	β_3_^(*a*)^	β_4_^(*a*)^	β_5_^(*a*)^	β_6_^(*a*)^	β_0_^(*a*)^	β_1_^(*b*)^	β_2_^(*b*)^	β_3_^(*b*)^	β_4_^(*b*)^	β_5_^(*b*)^	β_6_^(*b*)^	β_0_^(*b*)^
sign (value) in prior	–	–	–	–	–	–	96.46	+	+			–	–	2.21	+	+				–	–0.97

a Black cells indicate no sign constraints.

As shown in Table 5, the mean functions do not include nonlinear terms (e.g., *x* ^2^) because a linear relation was approximated between the concentration of a single color pigment^35^ and L*, a^*,^ and b* parameters. *f*_target_ selected a point with L*, a*, and b* values of [79.1, 2.40, 4.55]. The optimization trial, with a maximum of 20 iterations, was repeated 20 times, each starting from a randomly selected set of three data points.

### 2.5 Assessment of the Domain of Applicability for SP-GPR-SC

To clarify the proposed method’s scope and limitations, additional control experiments are conducted using benchmark mathematical Function No.1 (Table 1): parameter sensitivity to hyperparameters, the effect of using incorrect or inaccurate sign constraints, and the computational costs of MCMC sampling, as well as testing highly nonlinear mathematical Functions (No. 4–6 in Table 1). For these controls, 50 BO trials were conducted with EI as the acquisition function. The maximum number of iterations per trial was set to 20, and the mean DT value across 50 trials is summarized as a figure and used in the discussion.

#### 2.5.1 Sensitivity Evaluation for Parameters

Since BO (SP-GPR-SC) assumes a prior distribution for each parameter, the selection of hyperparameters for priors is examined by changing the hyperparameter values as shown in Table S1. In addition to the baseline condition, four levels other than the baseline value were evaluated for each of the four tested parameters, σ_*c*_, α_ρ_, β_ρ_, and σ_σ*z*_, resulting in a total of 17 conditions. In each modified condition, only the target parameter was changed, while the other parameters were fixed at their baseline values. Furthermore, the standard deviation of the normal distribution for β_k_ was varied from 1 to 128.

#### 2.5.2 Effects of Using Incorrect or Inaccurate Prior Knowledge

For Function No. 1, incorrect and/or inaccurate signs for the priors are tested as shown in Table 6. The focused linear components of Function No. 1 are 2*x*_1_ and 2*x*_2_, Case 1 in Table 6 is identical to the baseline condition for Function No. 1 used in Section 2.4.1. Cases 2 to 4 are conditions in which one or both signs are inconsistent with those of F­(**x**)’s linear components, and Case 5 represents a partially correct sign. In Cases 4 and 5, no sign constraint was imposed on β_2_ to represent a situation in which prior knowledge is available only for part of the coefficients.

**Table 6:** Sign Settings for Prior Distributions for Function No. 1^a^ ^,^ ^b^

case	β_1_	β_2_
1	+	+
2	–	+
3	–	–
4	–	
5	+	

a Coefficient β_0_ is omitted due to no constraint being given to the parameter.

b Black cells indicate no sign constraints.

#### 2.5.3 Effects of Data Dimensionality and Size on Computational Cost

To evaluate the computational cost of Bayesian estimation (MCMC sampling), computation time is measured as the feature dimension D and the number of training data *N* used to build the GP model were varied. In this analysis, the Stan parameters are set as iter = 300, warmup = 100, and chains = 1, and a posterior distribution with 200 samples is computed for each condition. Computation time is monitored over the ranges *D* = 2–66 and *N* = 23–368. *D* is controlled by introducing Fourier features added to the model’s polynomial. Specifically, sin­(2π*kx*) and cos­(2π*kx*) (*k* = 1, 2,...) are sequentially added for the input variables *x*_1_ and *x*_2_. Together with the original *x*_1_ and *x*_2_, these features formed the feature vector Φ_D_(**x**) as follows.

$$\Phi_{D}(x)=[x_{1},x_{2},\sin(2\pi x_{1}),\cos(2\pi x_{1}),\sin(2\pi x_{2}),\cos(2\pi x_{2}),\cdot \cdot \cdot {]}^{T}$$ 23

Φ_*D*_(**x**) is the input of the ARD kernel, and the mean function is a linear combination of Φ_D_(**x**).

## 3 Results and Discussions

### 3.1 BO Trials for Benchmark Mathematical Functions

#### 3.1.1 Comparison among BO Methods

For each of the three benchmark mathematical functions introduced in Section 2.4.1, 50 BO trials were conducted to identify the maximum functional value. For each trial, the maximum number of iterations was 20, and the transition of DT values, which represent the distances to the target value, was monitored. The acquisition function used in this case study was EI. The transitions of the average of DT values (DT_average_) over the 50 trials are reported in Figure 3 for the cases where the governing functions contained dominant polynomial terms. For all three tasks, the SP-GPR-SC method approached the target values with the fewest iterations, while the MLR-based optimization method exhibited the worst performance at DT_average_ on the 20th iteration. The SP-GPR without sign constraints (SP-GPR) and the zero-mean GP model exhibited BO performance in between. Furthermore, a statistical hypothesis test showed SP-GPR-SC outperformed MLR, GPR, and SP-GPR in DT values at the 5% significance level based on the Wilcoxon rank-sum test at the fourth iteration. The results shown above were consistent when PI was used as the acquisition function instead of EI, as shown in Figure S3 for the No. 1 function. Thus, in the following section, only results for EI are reported.

**Figure 3:**
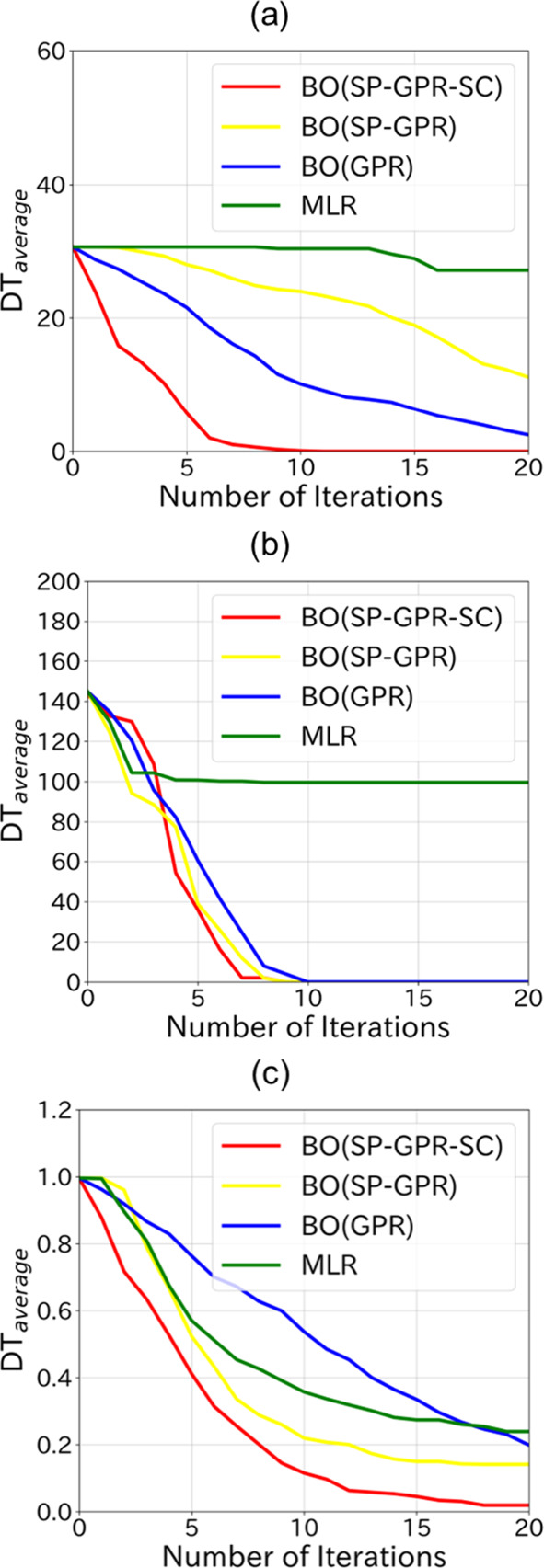
Transitions of the average of DT values over the 50 trials for (a) Functions No. 1, (b) No. 2, and (c) No. 3.

Incorrect linearity prior knowledge degraded BO performance on multimodal and highly nonlinear benchmark functions, as shown in Figure 4. For Functions No. 4, 5, and 6, BO using SP-GPR-SC performed worse than standard BO (GPR) in terms of DT_average_, although it overall performed better than MLR. This suggests that an appropriate assumption about prior knowledge is key to efficient BO search, which is further discussed in Section 3.4.

**Figure 4:**
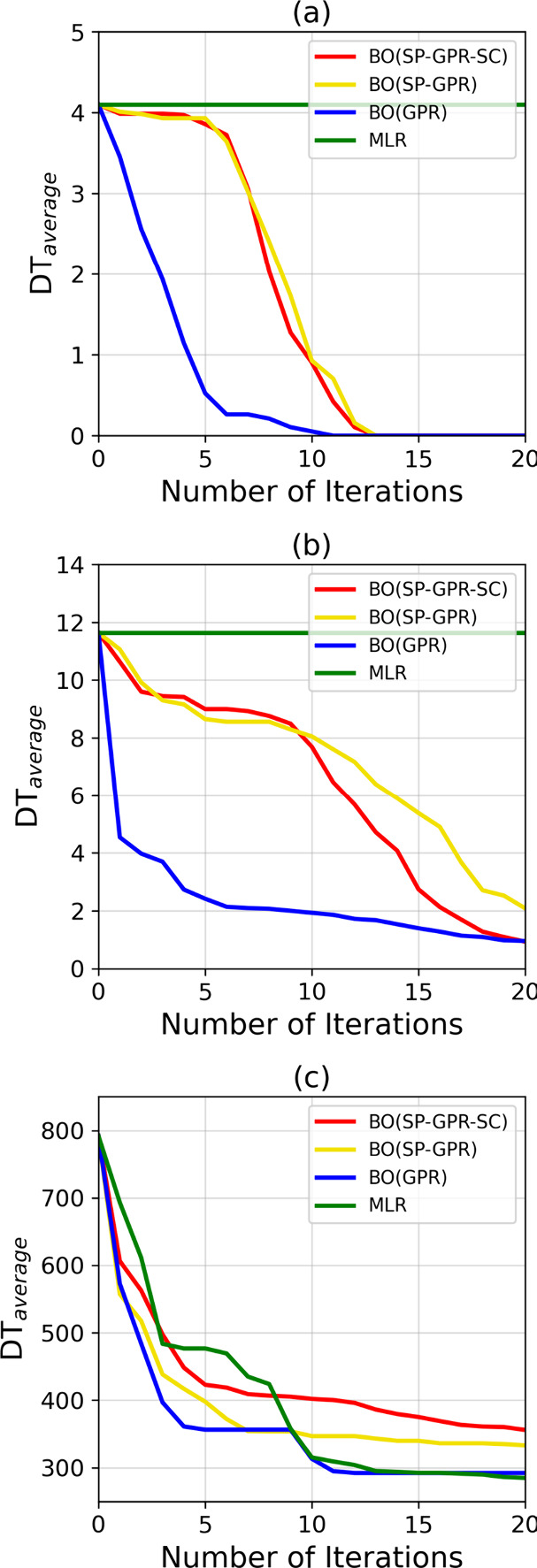
Transitions of the average of DT values over the 50 trials for (a) Functions No. 4, (b) No. 5, and (c) No. 6.

#### 3.1.2 Example: Sampled x in a BO Trial for Function No. 1

To better understand differences among BO methods, sampled points for the four BO methods are visualized on the search space at iterations 1, 3, 5, 8, 11, 14, and 17 for function No. 1 (Figure 5), where the BO (SP-GPR-SC) method is in Figure 5a, the BO (SP-GPR) in Figure 5b, the BO (GPR) in Figure 5c, and the MLR-based optimization in Figure 5d. Each panel also shows the contour as predicted surface of the *y* value by the model. In Figure 5, blue dots represent sampled points, while red dots represent initial points. Among the four methods, BO­(SP-GPR-SC) produced a contour at iteration 8 that was similar to that of the ground-truth function (Figure 2), achieving the target value *f*_target_ as well. BO­(GPR) converged to a contour equivalent to the ground-truth function and reached *f*_target_ at iteration 17. On the other hand, BO­(SP-GPR) and MLR failed to capture the upward trend in *f*_target_ across the search space, leading to incorrect exploration of the lower half of the search space. The effect of introducing the sign constraints in SP-GPR, i.e. SP-GPR-SC was demonstrated by its ability to grasp the search space and reach *f*_target_ in the early iterations (Figure 5a).

**Figure 5:**
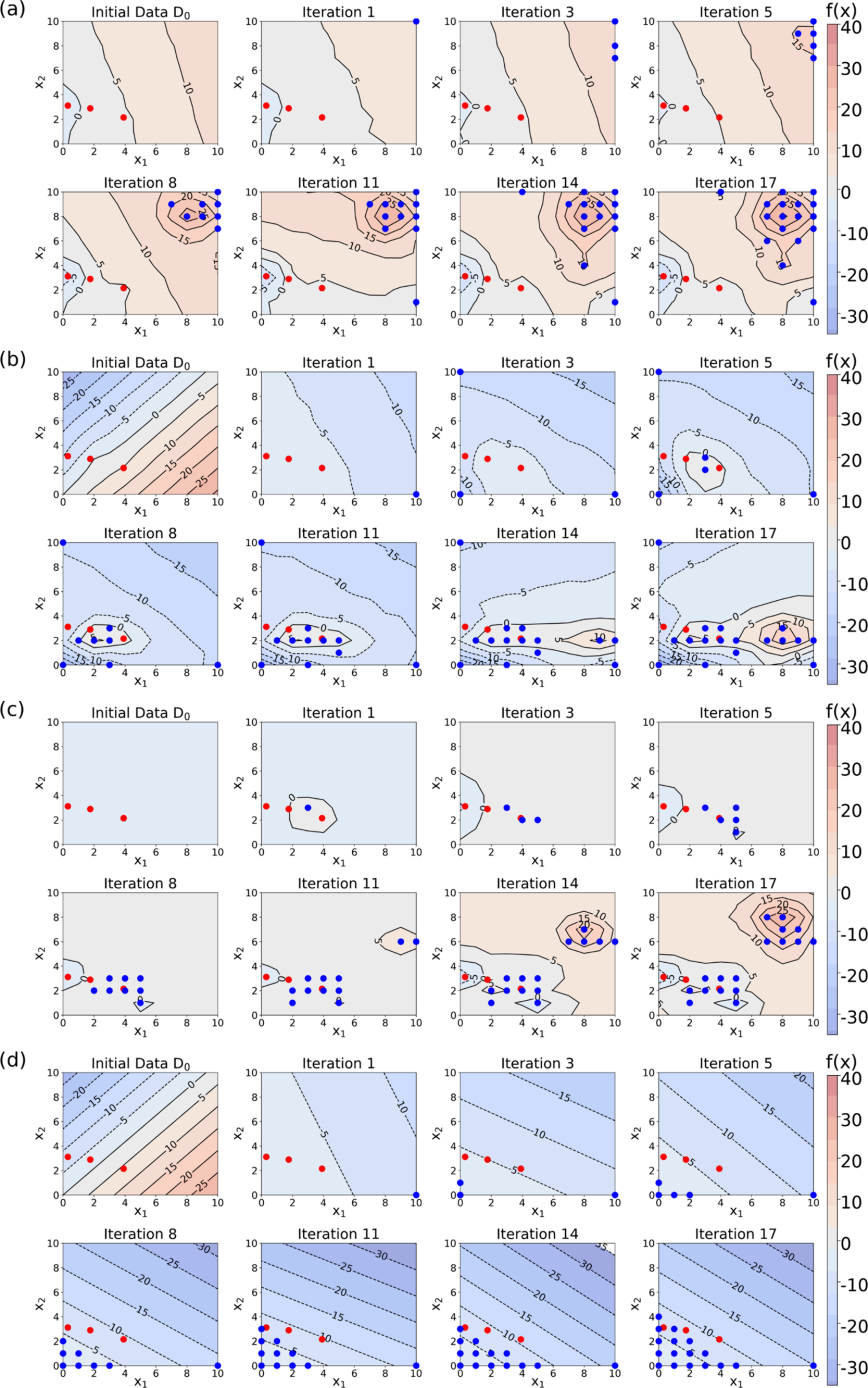
Transition of sampled points during the BO trials for Function No.1 by (a) BO (SP-GPR-SC), (b) BO (SP-GPR), (c) BO­(GPR), and (d) the optimization based on MLR. Red dots represent the initial data set, and blue dots represent the sampled points during the BO trial up to the iteration specified in the figure title. The contours and surfaces in each panel represent the model’s predicted mean *y* values across iterations.

#### 3.1.3 Posterior Distribution Transition for the Coefficients of the Linear Structure in the Case of Function No. 1

SP-GPR-SC assumed a prior for the coefficients β_1_ and β_2_ of the linear structure of the mean function of GPR, and the posterior (eq 14) was estimated using MCMC sampling. The posterior distributions for BO (SP-GPR-SC) and BO (SP-GPR) over the number of iterations for Function No. 1 are shown in Figures 6a and b, respectively. A sign constraint for each variable was introduced as a truncated normal distribution of prior, which was also applied to posteriors as shown in Figure 6. Since the linear structure of Function No. 1 is 2*x*_1_ + 2*x*_2_, parameters β_1_ and β_2_ are expected to be 2. For SP-GPR-SC, as shown in Figure 6a, the point estimates of β_1_ and β_2_ after iteration 14 were approximately 0.8, which is relatively close to 2, demonstrating a relatively good representation of the linear structure. On the other hand, as shown in Figure 6b, the point estimate of β_2_ for SP-GPR was negative, indicating a difference in the ability of the two methods to capture the appropriate linear structure.

**Figure 6:**
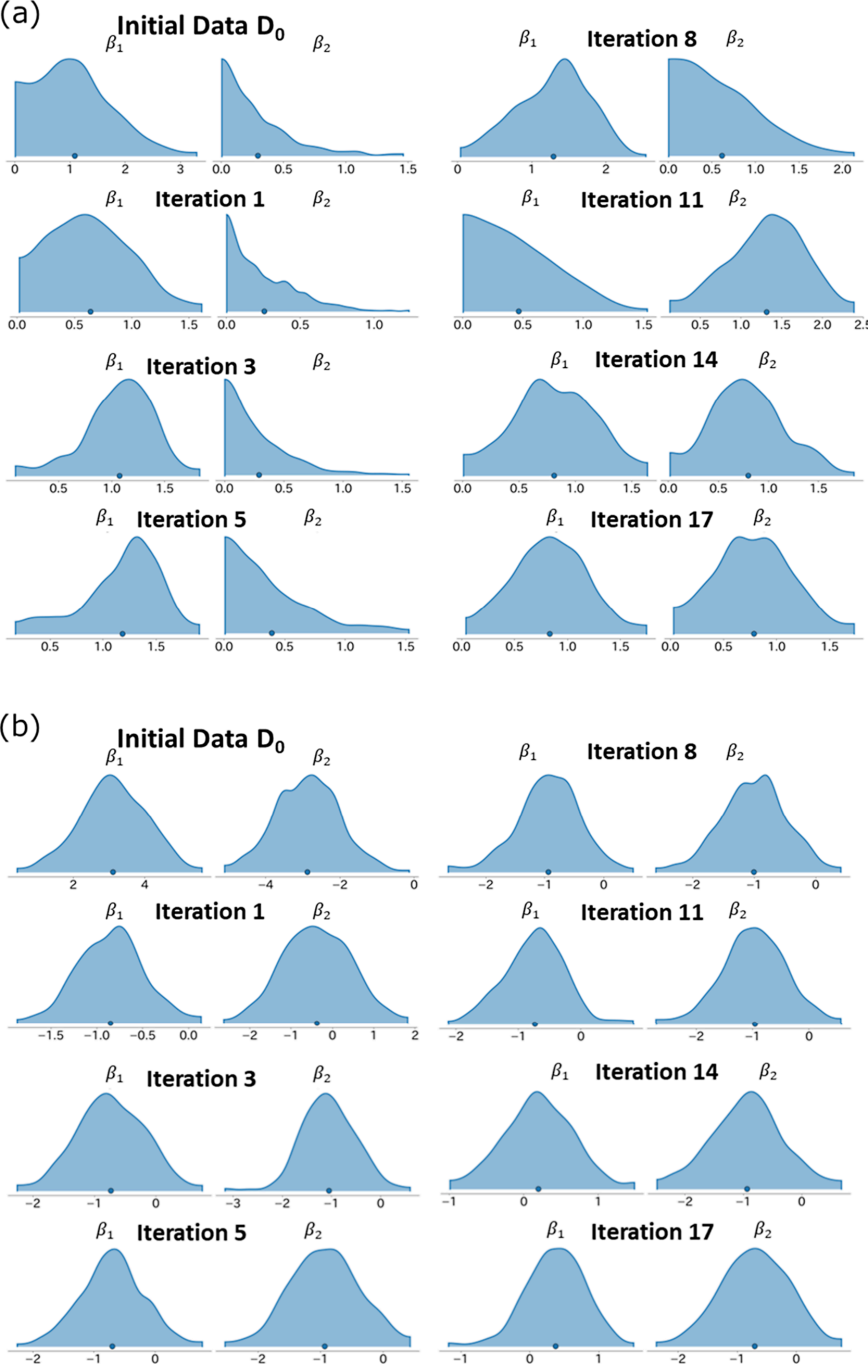
Posterior distribution transition of the coefficients of the linear structure in the GPR models for (a) BO (SP-GPR-SC) and (b) BO (SP-GPR).

Basically, Function No. 1 has linear and nonlinear terms, and the exact linear structure would not be obtained due to the presence of the nonlinear terms. However, in this study, the coefficients of the linear structure were estimated with relatively high accuracy.

### 3.2 BO Trials for the BP Data Set

#### 3.2.1 Comparison among BO Methods for the BP Data Set

The DT_average_ transitions using two nonredundant and nine redundant variables are shown in Figures 7a,b, respectively. While BO­(GPR) exhibited poor convergence, especially when nine redundant variables were used, BO­(SP-GPR-SC), BO­(SP-GPR), and MLR, which utilize linear structures, were confirmed to approach the minimum DT_average_ quickly. In particular, BO­(SP-GPR-SC) exhibited the fastest convergence to the minimum DT_average_ throughout the BO trials, regardless of the independent variable types. Unlike in the benchmark function case study, the MLR-based optimization method performed sufficiently. Statistical hypothesis testing also showed that SP-GPR-SC outperformed MLR, GPR, and SP-GPR in DT values at the 5% significance level in the Wilcoxon rank-sum test at the sixth iteration.

**Figure 7:**
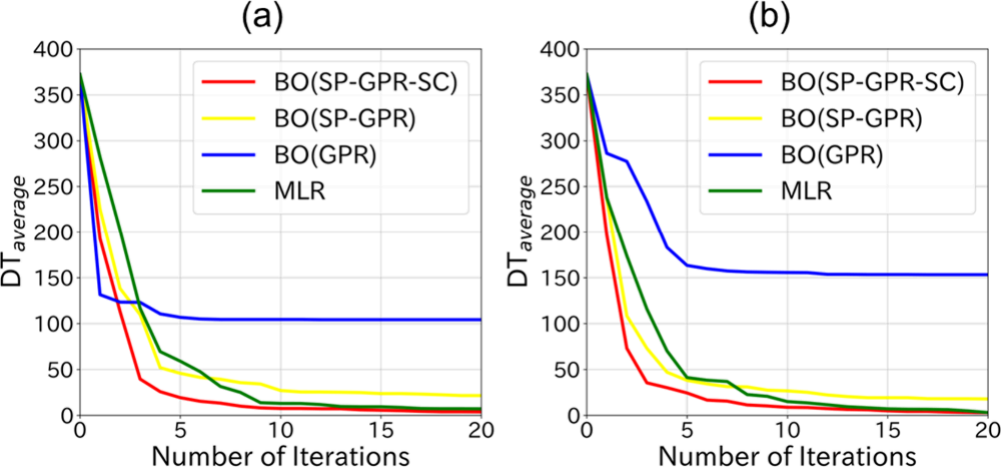
Transition of DT_average_ for the four methods: BO (SP-GPR-SC), BO (SP-GPR), BO (GPR), and the MLR-based optimization using (a) nonredundant and (b) nine redundant variables.

#### 3.2.2 Example: Sampled *x* in a BO Trial for the BP Data Set

The BP search aimed at identifying a compound with a *f*_target_ of 417.48 K was examined as an example BO trial using nine independent variables (the redundant variable case). Each trial is visualized in the projected search space on the PCA axes. PC1 and PC2 in the PCA are calculated loading vectors derived from all **x**, and the search space is represented by BP contours. The contours are drawn using BP values at each grid point, computed by interpolating all available data. Figure S6 shows the projected search space of PCA. The first and second principal component axes accounted for 49.4 and 26.6% of the total variance, respectively. In a BP search, in addition to quickly approaching the *f*_target_, accurately predicting BP values in the search space is important. Figure 8a–d shows the PCA contours and sampling points created using the BPs predicted by the four methods: BO (SP-GPR-SC), BO (SP-GPR), BO (GPR), and MLR-based optimization, at iterations 3, 8, and 17, respectively. In Figure 8, the points marked with green stars indicate the target, and the red plots indicate initial points (BPs: 164.56, 184.69, and 189.69 K). Comparing the predicted surfaces in PCA space among the four methods, BO (SP-GPR-SC) obtained experimental points that were close to the actual contours and *f*_target_ as early as iteration 3 (Figure 8a). BO (SP-GPR) began to resemble the actual contours from iteration 8 onward and approached *f*_target_ by iteration 17 (Figure 8b). BO (GPR) failed to obtain an appropriate contour during the entire iterations (Figure 8c). MLR obtained complex contours at iteration 8, indicating an unstable model (Figure 8d). The effect of introducing sign constraints into SP-GPR could be understood as forcing the prediction surface toward the prior knowledge, which was dominant during the early stage of BO iterations.

**Figure 8:**
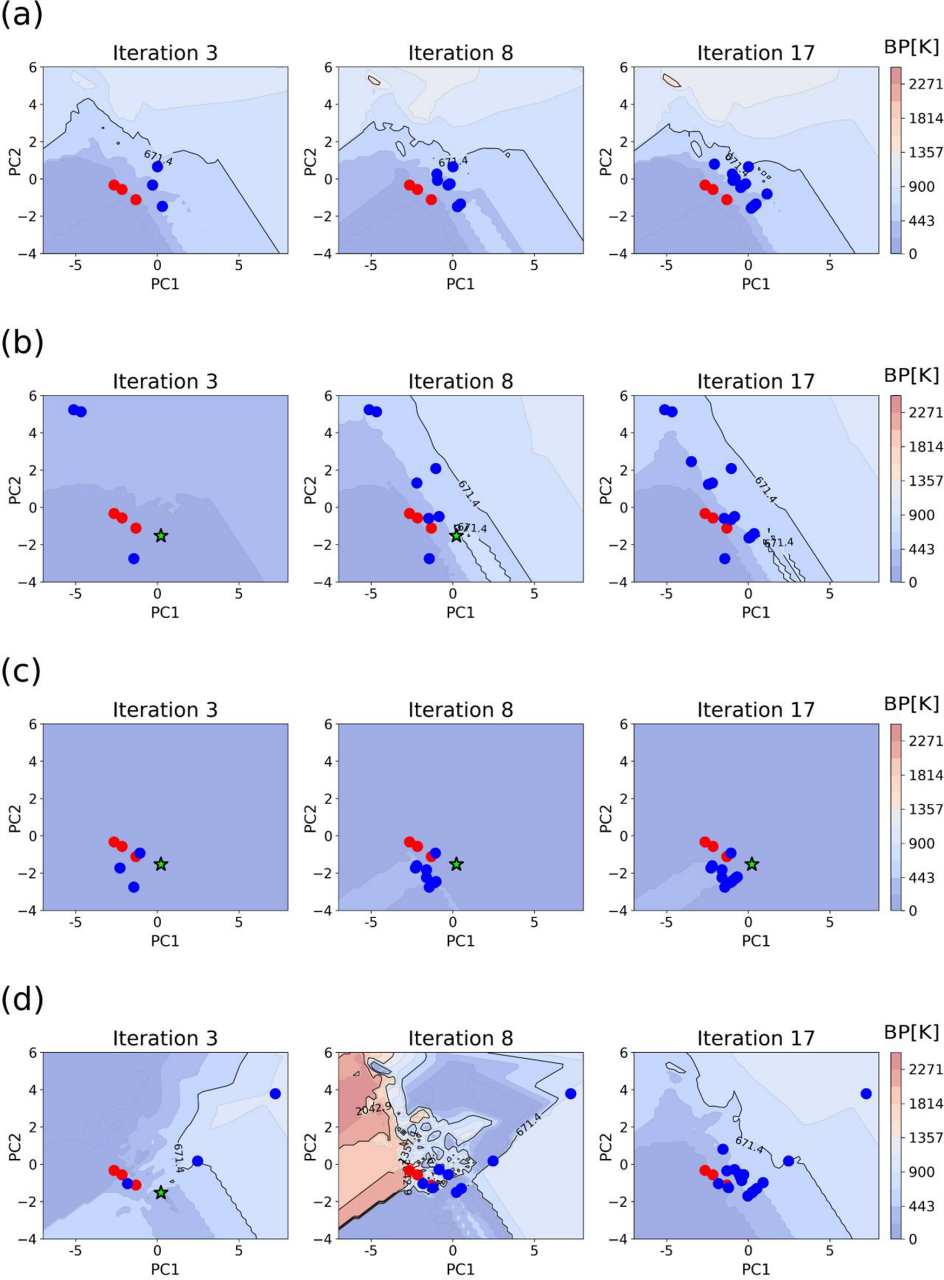
Sampled compounds for the BP data set through optimization with (a) BO (SP-GPR-SC), (b) BO (SP-GPR), (c) BO­(GPR), and (d) MLR. Blue dots represent sampled points, red dots initial points, and the green star *f*_target_.

#### 3.2.3 Transition of Regression Coefficients for the BP Data Set

Figure 9 shows the transition of the posterior distributions for the linear structure of BO (SP-GPR-SC) and BO (SP-GPR) when using nine independent variables (redundant variables), focusing on the linear parameters β_8_ (topological polar surface area) and β_9_ (log­(molecular weight)) at iterations 1, 3, 5, 8, 11, 14, and 17. Because the sign constraint was introduced as a truncated normal distribution for BO (SP-GPR-SC), the distribution for β_8_ remained positive at the 17th iteration, while BO (SP-GPR) generated a posterior for β_8_ centered around 0 from iteration 5 onward, indicating ignoring the variable in the regression system. Regarding posterior convergence, for BO (SP-GPR-SC) after the 11th iteration, point-estimated parameters β_8_ and β_9_ fell within the ranges of 8.0–8.5 and 710–730, respectively, indicating convergence (Figure 9a). The point estimate parameters β_8_ and β_9_ for BO (SP-GPR) converged within the ranges of −0.1 to 0 and 495 to 505, respectively, after the 11th iteration (Figure 9b). In terms of convergence speed, BO­(SP-GPR-SC) approached the final converged value as early as iteration 3, while BO­(SP-GPR) tended to approach the final converged value from iteration 5 onward, demonstrating its superiority.

**Figure 9:**
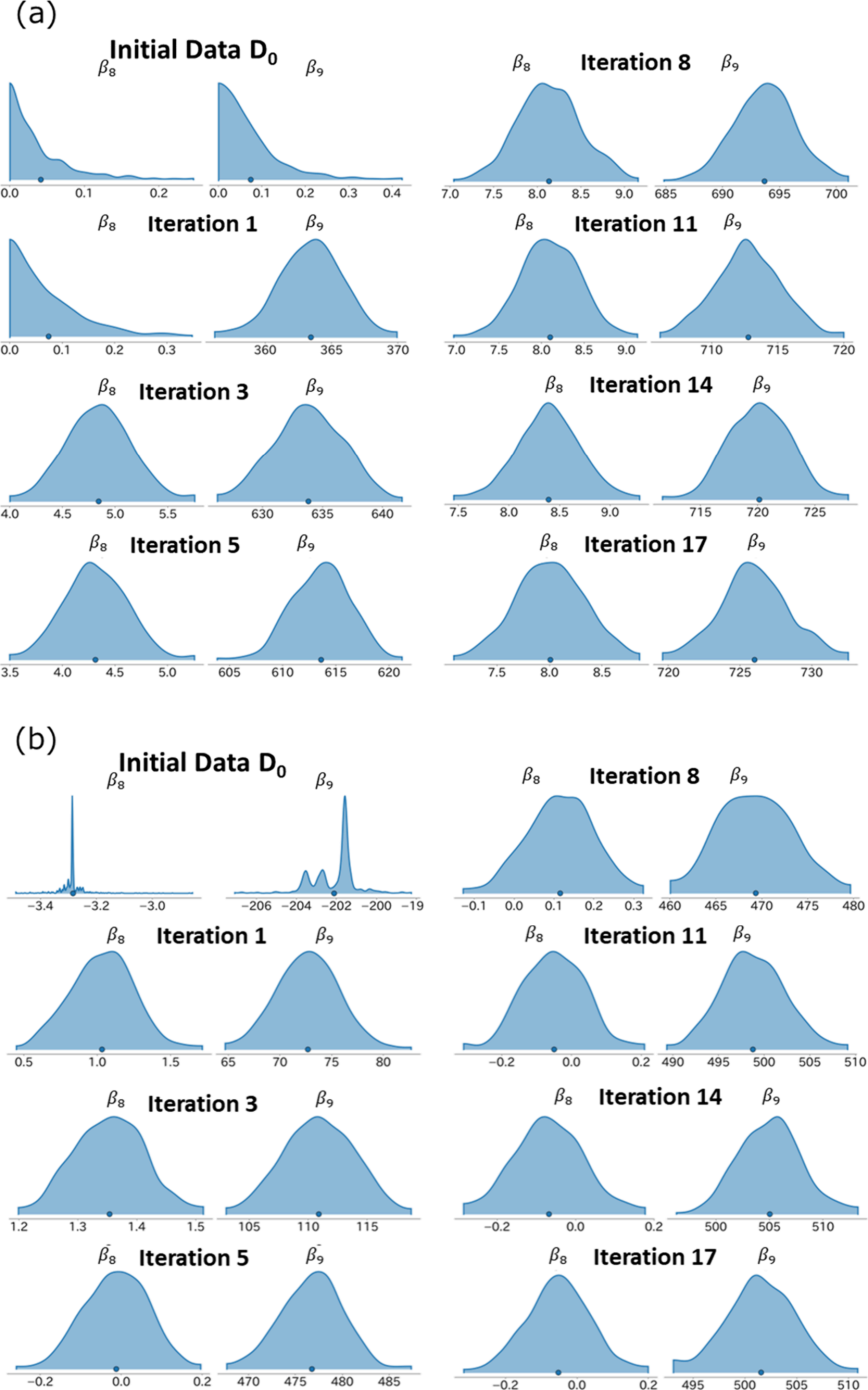
Transition of posterior distributions of β_8_ and β_9_ for topological polar surface area and log­(molecular weight), respectively. (a) BO (SP-GPR-SC) and (b) BO (SP-GPR) are shown.

### 3.3 BO Trials for the Pigment Data Set

#### 3.3.1 Comparison among BO Methods

Twenty BO trials were conducted for each method to identify the ratios that retrieved the predefined color (coded by L*, a*, and b*). The DT_average_ transitions across 20 trials are reported in Figure 10. The proposed BO (SP-GPR-SC) method approached *f*_target_ more quickly than the other methods. Statistical hypothesis testing also showed that SP-GPR-SC outperformed MLR, GPR, and SP-GPR in DT values at a significance level of 5% in the Wilcoxon rank-sum test at the sixth iteration.

**Figure 10:**
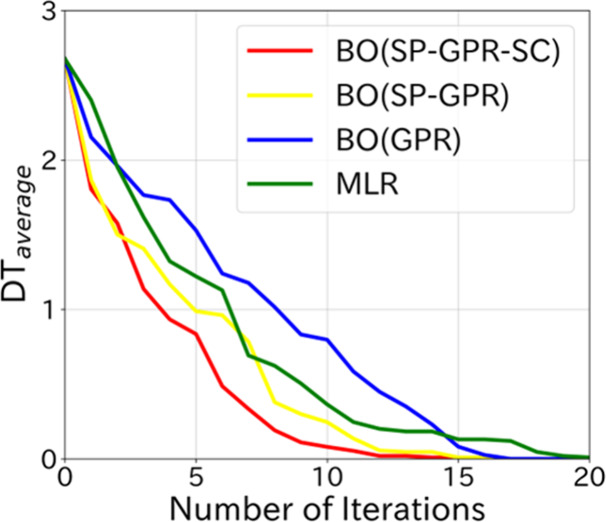
Transition of DT_average_ values for the four methods: BO (SP-GPR-SC), BO (SP-GPR), BO (GPR), and the MLR-based optimization using the pigment data set.

#### 3.3.2 Example: Sampled x in a BO Trials for the Pigment Data Set

Figure S6 shows the L* contours on the PCA surface constructed using the entire pigment data set. The variance contributions of PC1 and PC2 were 21.6 and 20.6%, respectively. Panels in Figure 11 show PCA surface plots of the sampling points at iterations 3, 8, and 17 for the four methods (BO (SP-GPR-SC), BO (SP-GPR), BO (GPR), and MLR-based optimization), along with contours constructed using the predicted L* values of the models built by each method. The point marked with a green star in Figure 11 was the target (L* = 79.13), and red points represent the initial data set. Comparing the predicted surfaces of the four methods, BO­(SP-GPR-SC) and BO­(SP-GPR) showed similarity to the actual contours as early as iteration 3 (Figures 11a,b), whereas BO­(GPR) showed unclear contours until iteration 17 (Figure 11c), and MLR obtained complex contours at iteration 8, resulting in an unstable model (Figure 11d). Furthermore, in terms of approaching the *f*_target_, BO­(SP-GPR-SC) and BO­(SP-GPR) showed superiority, reaching the *f*_target_ at iteration 8. The effectiveness of introducing sign constraints into SP-GPR was demonstrated by its rapid exploration of the search space and its proximity to the *f*_target_.

**Figure 11:**
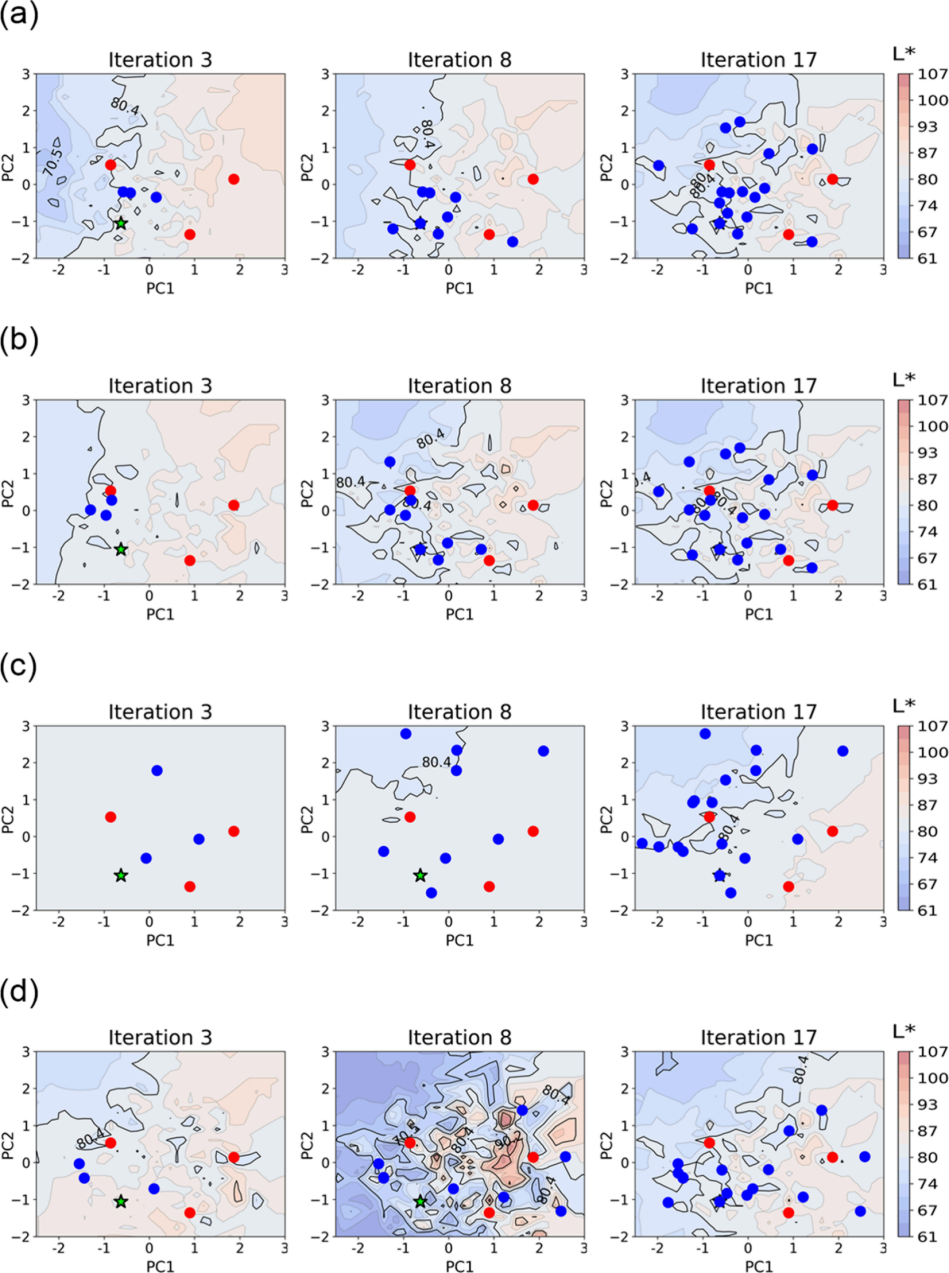
Sampled data points for the pigment data set through optimization with (a) BO (SP-GPR-SC), (b) BO (SP-GPR), (c) BO­(GPR), and (d) MLR. Blue dots represent sampled points, red dots initial points, and the green star *f*_target_.

#### 3.3.3 Transition of Regression Coefficients for the Pigment Data Set

Figure 12 shows the transition of the posterior distributions of the six coefficients for L*, aiming at identifying a pigment combination for L*, a*, and b* = 79.1, 2.40, and 4.55, respectively. The sign constraints were introduced as truncated-normal priors; the posterior inherited the same feature by Bayes’ theorem. From iteration 14 onward, both BO (SP-GPR-SC) and BO (SP-GPR) generally converged to similar point estimates for β_1_ through β_6_, with no significant difference in convergence across methods. Before iteration 8, BO (SP-GPR-SC) consistently produced negative point estimates on the initial data, whereas BO (SP-GPR) produced positive estimates for multiple βs. Although the difference between the two methods diminished as the optimization cycle progressed (in this case, exceeding 8 iterations), SP-GPR-SC contributed to identifying near-optimal solutions in the early stage.

**Figure 12:**
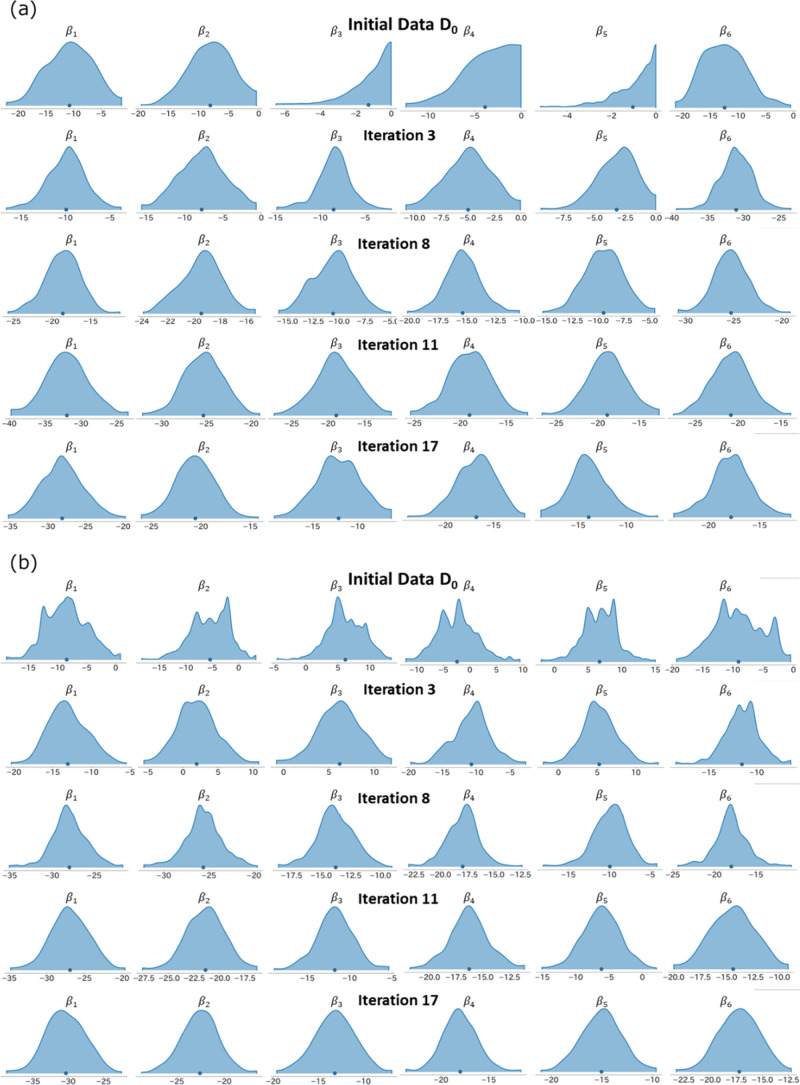
Transitions of posterior distributions of the coefficients of the linear structures for L* of (a) BO (SP-GPR-SC), (b) BO (SP-GPR).

### 3.4 Assessment of BO (SP-GPR-SC)

#### 3.4.1 Hyperparameter Sensitivity

For benchmark Function No. 1 in Table 1, the transitions of DT_average_ under different hyperparameters are shown in Figure 13. One hyperparameter was varied while the others were kept at the baseline values. Stable performance across hyperparameter changes was observed for σ_*c*_: 1–4 for *c*, α_ρ_: 3–8 and β_ρ_: 2.5–10 for ρ_*d*_, and σ_σ*z*_: 0.25–1 for σ_*z*_, showing that BO (SP-GPR-SC) maintained good search performance over a relatively wide range of prior distribution settings around the baseline values used in this study. The shape of DT_average_ was almost identical when the standard deviation of the prior distribution of coefficient β_*k*_ was in a range of 2–128 (Figure S7), implying that a relatively large σ_β*k*_ worked for BO­(SP-GPR-SC).

**Figure 13:**
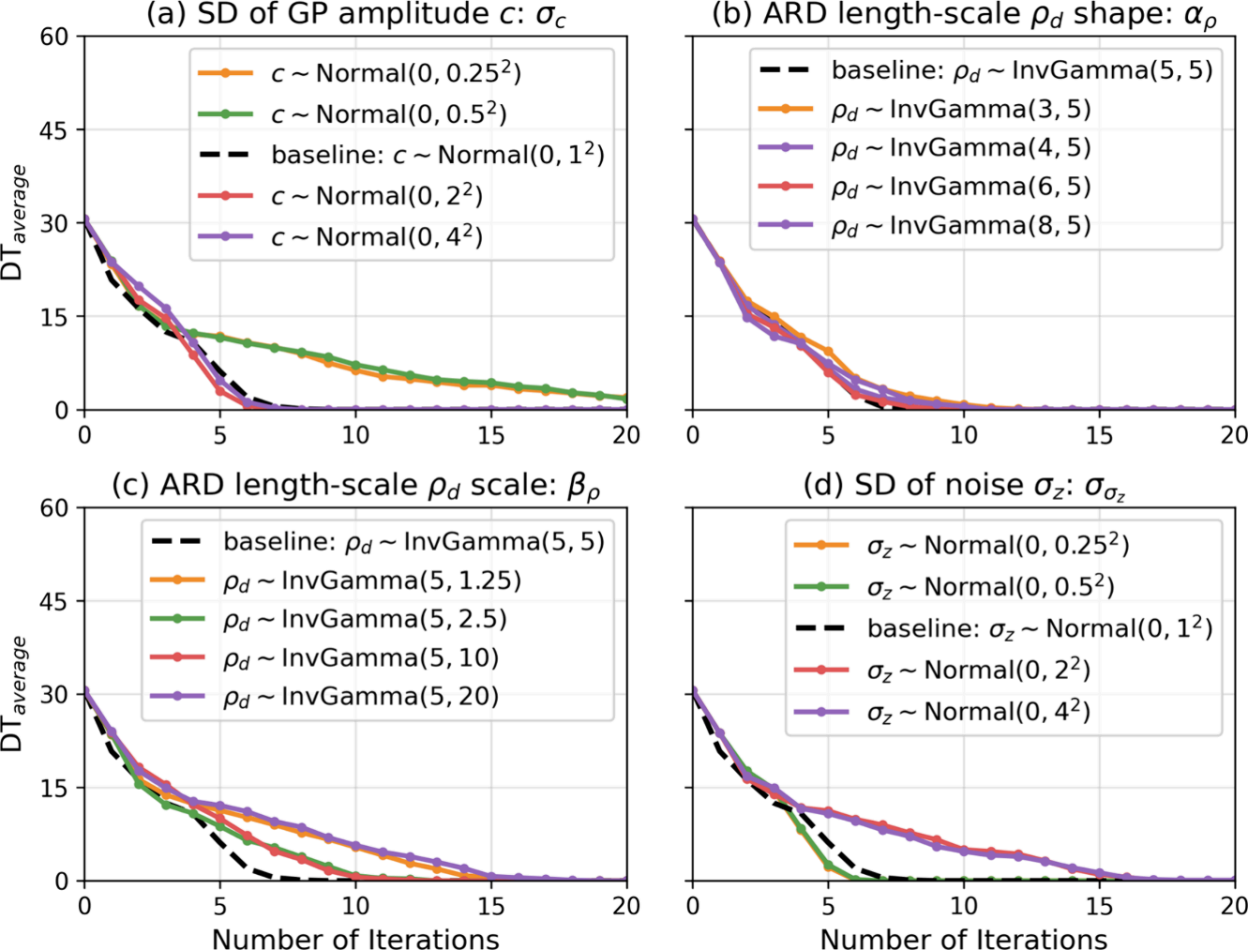
Sensitivity analysis for ARD-kernel hyperparameters. (a) standard deviation σ_*c*_ of the normal distribution for c; (b), (c) scale parameter β_ρ_ and shape parameter α_ρ_ of the inverse-gamma distribution for ρ_*d*_; and (d) standard deviation σ_σ*z*_ of the normal distribution for σ_*z*_.

#### 3.4.2 Incorporating Incorrect or Inaccurate Knowledge on BO (SP-GPR-SC)

Incorrect or partially correct sign constraints shown in Table 6 were set, and BO trials for Function No. 1 were conducted. DT_average_ transitions are summarized in Figure 14. The correct priors achieved converged DT_average_ close to 0 (Cases 1 and 5), superior to searches using incorrect constraints (Cases 3 and 4). The partially correct and incorrect signs (Case 2) yielded a DT_average_ transition between those of the correct and incorrect constraints. These results suggest that sign constraints can assist the search direction when the knowledge is correct, while incorrect knowledge can impair search efficiency.

**Figure 14:**
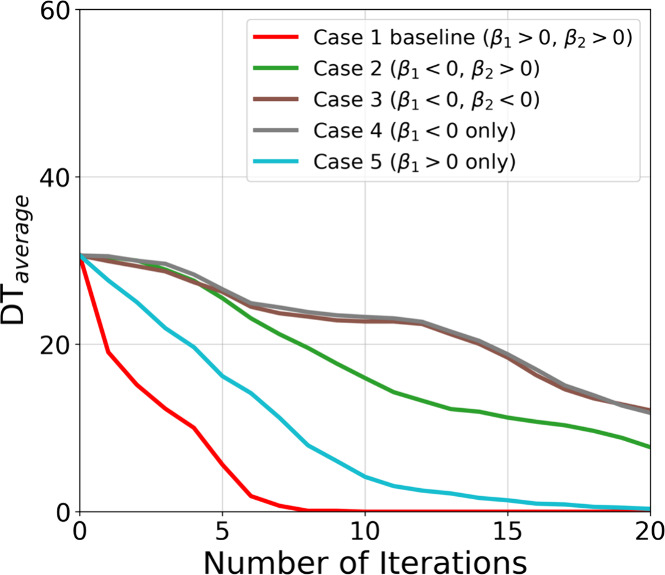
Transition of DT_average_ for the incorrect or partially correct sign constraints. The signs for the priors are shown in Table 6, where the correct sign constraint was Case 1, while other conditions were incorrect or partially correct.

#### 3.4.3 Computational Cost of BO (SP-GPR-SC)

As shown in Figures 15a,b, sampling time increased as the feature dimensions D or the number of training data N increased. When a model was trained on 92 data points, a feature dimension of 40 was already beyond practical handling due to its long sampling time. In BO (SP-GPR-SC), the GP model parameters are treated in a fully Bayesian manner; therefore, many posterior samples are generated via MCMC, and predictive distributions and acquisition functions are evaluated for each data point. Consequently, as the number of training samples or feature dimensions increases, the computational burden grows, imposing practical limitations when applying the method to extremely high-dimensional data or to large training sets.

**Figure 15:**
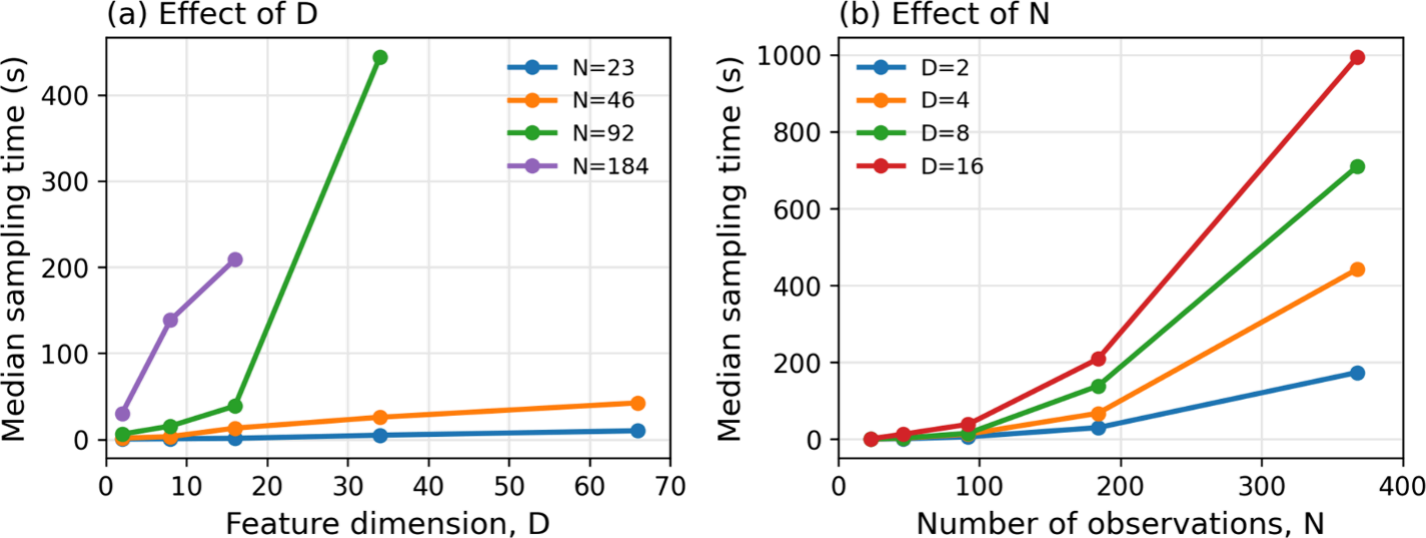
Stan sampling time of BO (SP-GPR-SC). Stan sampling times were plotted against the number of feature dimension *D* (a) and the number of training data (observations) (b). Unplotted points: *D* = 66 for *N* = 92 and *D* = 34, 66 for *N* = 184 represent the calculation time was beyond 1000 s.

## 4 Conclusions

Here, we propose BO (SP-GPR-SC), a BO method that incorporates researchers’ knowledge as a sign constraint on the coefficients of a linear structure. The linear structure is used as the mean function in the GPR, and the posterior distributions of the coefficients are sampled using MCMC. These posterior distributions are used to evaluate an acquisition function to identify the next experimental conditions to be tested. The usefulness of the proposed method was demonstrated through three case studies: using mathematical benchmark functions, the BP data set, and the pigment data set.

Compared with previously proposed BO approaches, including BO (SP-GPR), the standard BO (GPR), and an MLR-based optimization method, BO (SP-GPR-SC) identified better solutions with fewer experimental trials in these retrospective evaluations. Furthermore, the response surface predicted by the GPR in the BO (SP-GPR-SC) method is more closely aligned with the ground-truth surfaces obtained by interpolating all data points. Although BO (SP-GPR) also converged to a similar response surface as iterations progressed, BO (SP-GPR-SC) demonstrated advantages in the early stages of iterations owing to the prior knowledge incorporated into the model, as evidenced by consistent signs of the coefficients.

Through rigorous control calculations, we also found that efficient optimization of BO (SP-GPR-SC) required correct knowledge of sign constraints. When the assumed signs of the coefficients were inaccurate, search efficiency deteriorated. Furthermore, the proposed method did not work for strongly nonlinear and multimodal governing functions, as it assumes a linear relationship between **x** and *y*. Additional computational time is required for MCMC sampling since the BO (SP-GPR-SC) is a fully Bayesian approach. This may limit the practical usage of the method when many training samples with high-dimensional features are used.

Although several limitations remain, including the computational time required for the Bayesian treatment, we hope the BO (SP-GPR-SC) approach will contribute to material and compound discovery through an interplay between researchers’ knowledge and a well-established BO method.

## Supplementary Material



## Data Availability

All data and code used in this study, including BO­(SP-GP-SC), are publicly available in the GitHub repository at https://github.com/Hiroshi-Aoki164/BO-SP-GP-SC

## Notes

Boiling point (BP) histogram (Figure S1); L*, a*, and b* Distributions (Figure S2); comparison of DT average values between EI and PI as an acquisition function (Figure S3); explanation of L*a*b* simulator (Section S1); schematic of XYZ derivation (Figure S4); conditions used for the sensitivity analysis of the prior distributions for the GP kernel (Table S1); PCA-projected search spaces for the BP and pigment data sets (Figures S5 and S6); and sensitivity analysis of the standard deviation of the prior distribution for coefficient β_*k*_ (Figure S7) (PDF)
