# Supplementary material for: Explicitly Incorporating Prior Knowledge into Bayesian Optimization for Materials Design
Source: ACS Omega. 2026 Jul 10;11(29):43492–509. doi: 10.1021/acsomega.6c02171 (PMC13425488; doi:10.1021/acsomega.6c02171)
Supplement: Supplementary file 1 [file ao6c02171_si_001.pdf]

Supporting Information for

Explicitly Incorporating Prior Knowledge into Bayesian Optimization for Materials Design

Hiroshi Aoki[1,2], Tomoyuki Miyao[3,1]*

[1]Graduate School of Science and Technology, Nara Institute of Science and Technology, 8916-5 Takayama-cho, Ikoma, Nara 630-0192, Japan

[2] Process Development Research Institute, Kao Corporation, 1334 Minato, Wakayama-shi, Wakayama 640-8580, Japan

[3]Data Science Center, Nara Institute of Science and Technology, 8916-5 Takayama-cho, Ikoma, Nara 630-0192, Japan

*Corresponding authors
Tomoyuki Miyao: E-mail: miyao@dsc.naist.jp

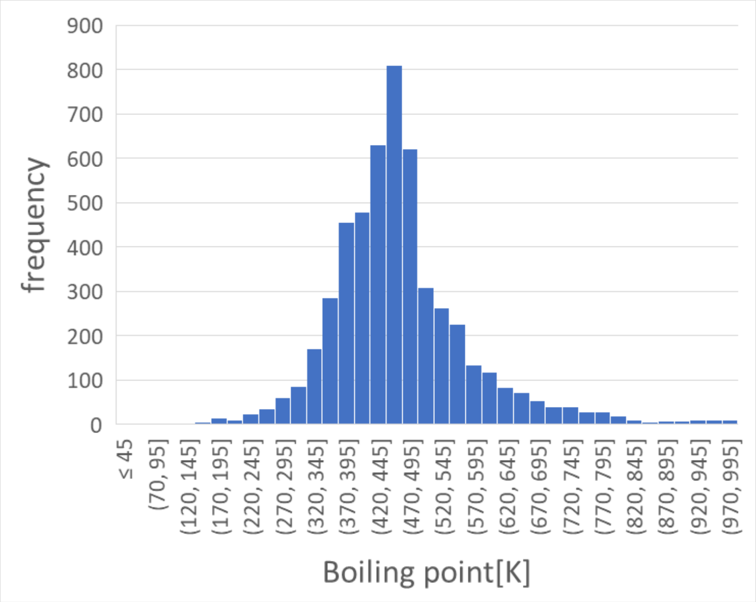

Figure S1 Boiling point (BP) histogram

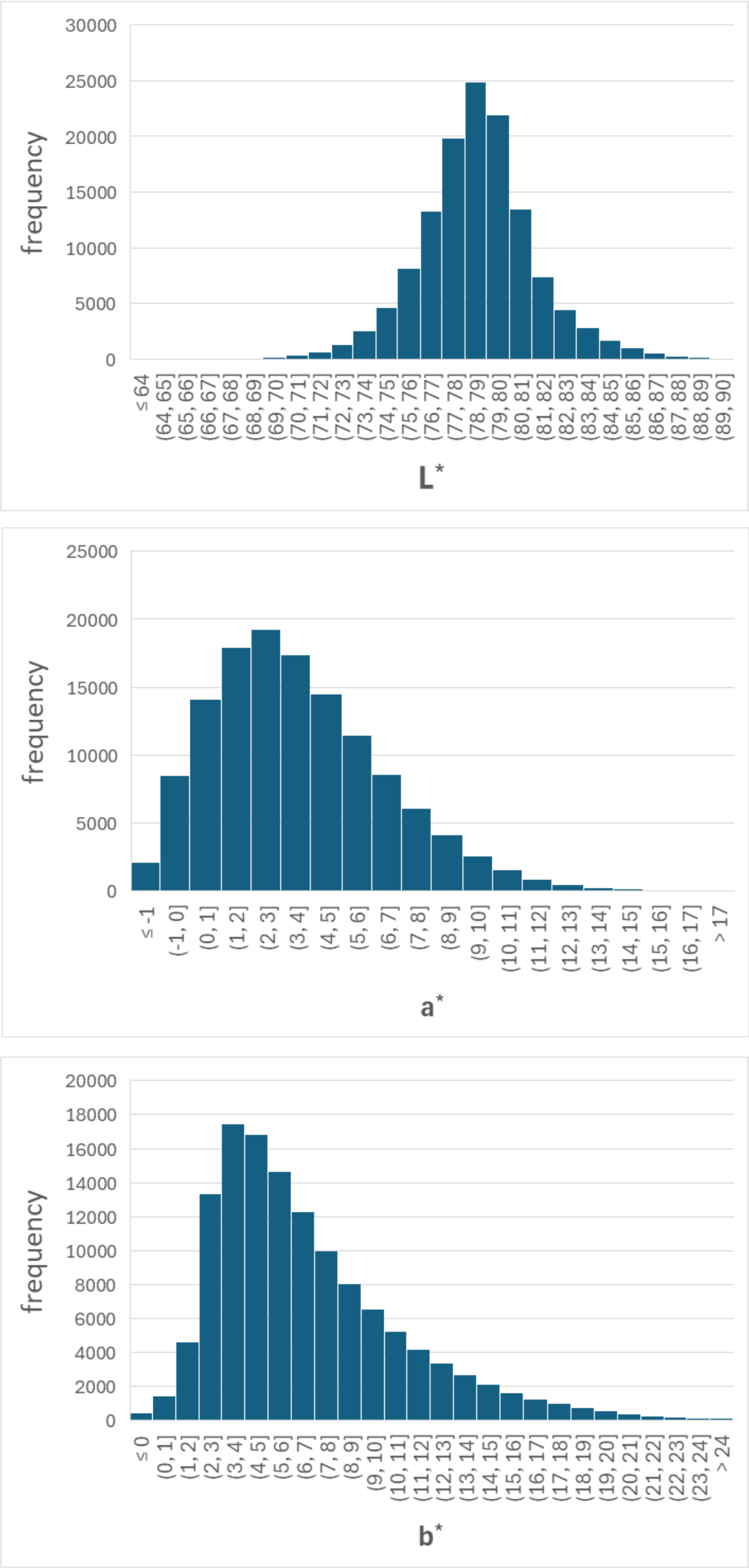

Figure S2 L^*, a^* and b^* Distributions

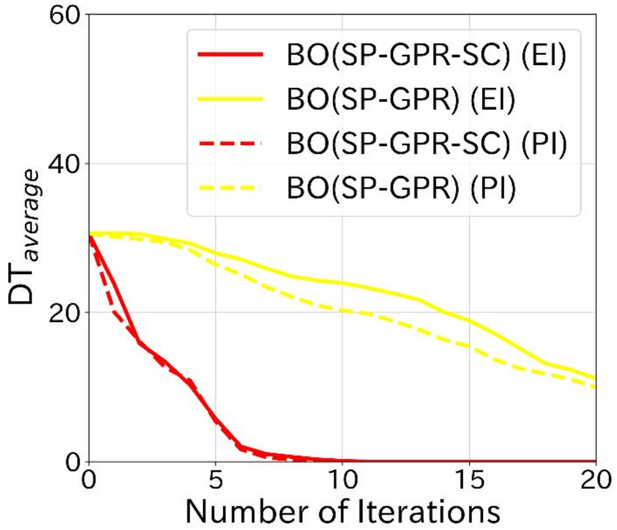

Figure S3. Comparison of DT average values between EI and PI as an acquisition function.

Section S1. $L^*a^*b^*$ simulator

The $L^*a^*b^*$ color space is one of the indices for color representation, which is also known as the CIE LAB color space. This color space was developed by the International Commission on Illumination (CIE) and allows the numerical representation of colors based on human vision.

$L^*a^*b^*$ is calculated from the XYZ color space using the following formula:

$$L^* = 116 \cdot f(Y/Y_n) - 16$$
$$a^* = 500 \cdot [f(X/X_n) - f(Y/Y_n)]$$
$$b^* = 200 \cdot [f(Y/Y_n) - f(Z/Z_n)]$$

Where $f(t)$ is defined as:

$$f(t) = \begin{cases} t^{1/3} & if\ t > (\frac{6}{29})^3 \\ \frac{1}{3} \cdot (29/6)^2 \cdot t + \frac{4}{29} & otherwise \end{cases}$$

X_n, Y_n, Z_n : Tristimulus values of a perfect reflecting diffuser.

The XYZ color space is calculated based on the characteristics of light source, the reflectance of the object, and chromaticity coordinates. The color of an object is perceived when light from the light source is reflected by its surface. The reflectance spectrum indicates how much an object reflects the light depending on wavelengths, and this spectral data can be used to calculate the XYZ values through the following formula.

$$X = k \int_{380}^{780} I(\lambda) R(\lambda) \bar{x}(\lambda) d\lambda$$
$$Y = k \int_{380}^{780} I(\lambda) R(\lambda) \bar{y}(\lambda) d\lambda$$
$$Z = k \int_{380}^{780} I(\lambda) R(\lambda) \bar{z}(\lambda) d\lambda$$

where, $k = \frac{100}{\int_{380}^{780} I(\lambda) \bar{y}(\lambda) d\lambda}$

$I(\lambda)$: illumination spectral distribution

$R(\lambda)$: sample spectral reflectance

$\bar{x}(\lambda), \bar{y}(\lambda), \bar{z}(\lambda)$: color-matching functions

where the parameter I(λ) is the D65 illuminant implemented in color science [1], and the parameters $\bar{x}(\lambda),\bar{y}(\lambda)\bar{z}(\lambda)$ are the CIE 1931 color-matching functions, 2 degree observer, also implemented in color science.

The schematics of deriving XYZ can be shown in **Figure S4.**

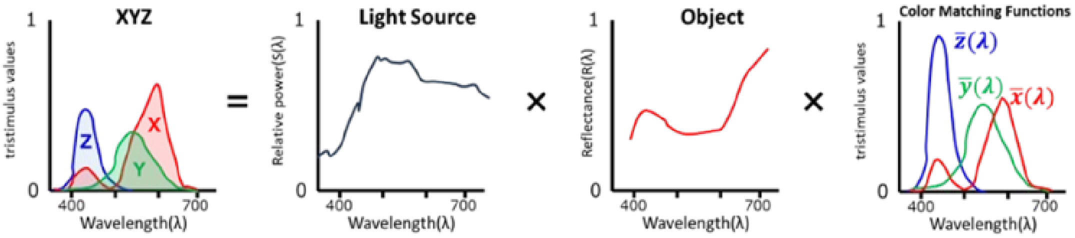

Figure S4. Schematic of XYZ derivation

Reflectance R is calculated based on the Kubelka-Munk theory, which is consisting of absorption coefficient K, scattering coefficient S, and film thickness l as follows:

$$R(l) = \frac{\frac{1}{R_\infty}(R_b - R_\infty) - R_\infty(R_b - \frac{1}{R_\infty})e^{Sl(\frac{1}{R_\infty} - R_\infty)}}{(R_b - R_\infty) - (R_b - \frac{1}{R_\infty})e^{Sl(\frac{1}{R_\infty} - R_\infty)}}$$

where,

$$R_\infty = 1 + \frac{K}{S} - \sqrt{\left(\frac{K}{S}\right)^2 + 2\frac{K}{S}}$$

R_∞ is the reflectance of a sample with an infinite thickness, and corresponds to the reflectance of a material on which light is completely diffused. Additionally, parameter K in the Kubelka-Munk theory is the absorption coefficient and expresses the degree to which the material absorbs light. Scattering coefficient S expresses the degree to which light is scattered within a material. The mixed composition parameters S and K can be expressed as follows using the S and K of the raw materials alone and the concentrations of each raw material:

$$S_{pred} = \sum S_i x_i$$

$$K_{pred} = \sum K_i x_i$$

where, S_i, K_i, and x_i correspond to the S and K of the *i-th* component of a material, and the concentration in the mixture composition, respectively. The $L^*a^*b^*$ values can be calculated by inputting a recipe (component ratios in the materials' mixture) of the color, as well as the parameters S and K for the components that make up the recipe, and the film thickness l, into the constructed simulator. The six coloring pigments described in the paper [2] are used as the materials that make up a recipe, and parameters S and K of each material are set to 100, as shown in Figure 6 of [2]. In this way, the simulator can calculate L^*, a^*, and b^* by providing the mixture composition of the six materials and the film thickness l to the simulator.

Table S1. Conditions for the sensitivity analysis of the prior distributions for the GP kernel. In ICvGamma(α_ρ, β_ρ), α_ρ and β_ρ denote the shape and scale parameters of the inverse-gamma distribution, respectively.

Target prior distribution	Changed parameter	Baseline value	Evaluated values
$c \sim \text{Normal}(0, \sigma_c^2)$	σ_c	1	0.25, 0.5, 2, 4
$\rho_d \sim \text{InvGamma}(\alpha_\rho, \beta_\rho)$	α_ρ	5	3, 4, 6, 8
$\rho_d \sim \text{InvGamma}(\alpha_\rho, \beta_\rho)$	β_ρ	5	1.25, 2.5, 10, 20
$\sigma_z \sim \text{Normal}(0, \sigma_{\sigma z}^2)$	$\sigma_{\sigma z}$	1	0.25, 0.5, 2, 4

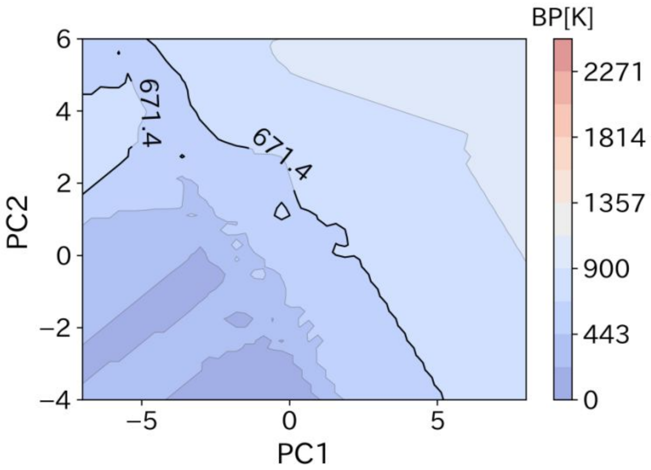

Figure S5. Search space projected onto the PCA axis

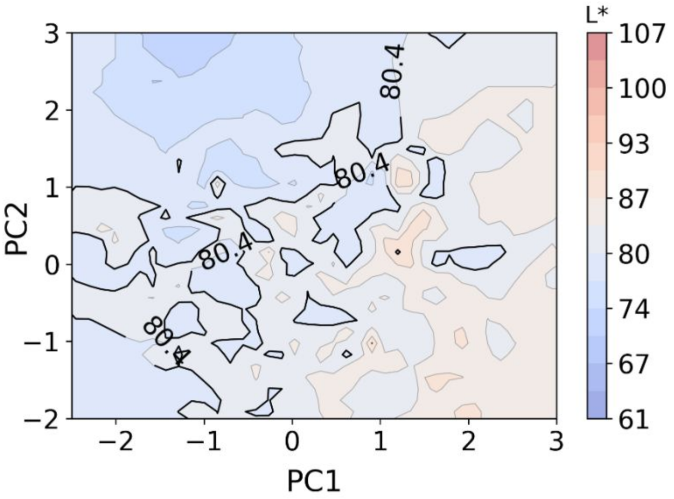

Figure S6. Search space projected onto the PCA axes.

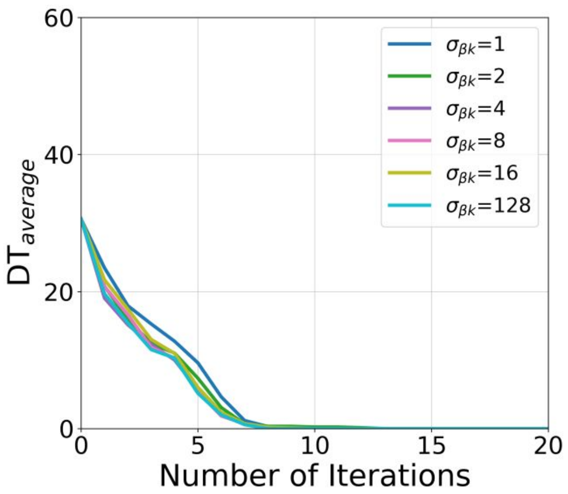

Figure S7. Sensitivity analysis of the standard deviation of the prior distribution for coefficient $\boldsymbol{\beta}_{\mathbf{k}}$.

References

1. T. Mansencal et al., Colour 0.4.4, Zenodo https://doi.org/10.5281/zenodo.10396329 (accessed 2026-06-24).
2. T. Aoyama, H. Chinen, T. Funatomi, Y. Mukaigawa, T. Morimoto, T. Oishi, J. Takamatsu, K. Ikeuchi, Validity of the Kubelka-Munk model for the analysis of ancient pigments, IPSJ SIG Technical Report, Vol. 2021-CG-184, No.14 / Vol.2021-DCC-29, No.14 / Vol. 2021-CVIM-227, No.14, pp.1–6, 2021. (in Japanese)
